# Antimicrobial Materials Used in Coating Dental Implant Surfaces: State of the Art and Future Prospectives

**DOI:** 10.3390/ma19020403

**Published:** 2026-01-19

**Authors:** Kazi Naziba Tahsin, Amin Rizkalla, Paul Charpentier

**Affiliations:** 1 School of Biomedical Engineering, Western University, London, ON N6A 3K7, Canada; 2 Department of Electrical & Electronics Engineering, Independent University, Bangladesh, Dhaka 1229, Bangladesh; 3 Department of Chemical & Biochemical Engineering, Western University, London, ON N6A 5B9, Canada

**Keywords:** dental materials, antimicrobial coatings, bioactive glass composites, antimicrobial polymers, metallic nanoparticles, antimicrobial peptides, dental implants, peri-implant infections

## Abstract

This review provides a comprehensive overview of dental materials that promote tissue healing while exhibiting antimicrobial properties. The focus is on materials that are biocompatible, bioactive, and non-toxic to host cells, with demonstrated bacteriostatic and bactericidal activities. Current advances in natural bactericides, antimicrobial polymers, and bioactive glass/polymer composites are summarized, along with techniques employed for surface modification and the coating of dental implants. Three major categories of antimicrobial coatings were identified: antibacterial phytochemicals, synthetic antimicrobial agents (including polymers and antibiotics), and metallic nanoparticles. Bioactive coatings were further examined to identify potential antimicrobial strategies within these materials, and existing research gaps were highlighted. A systematic literature search was conducted in PubMed, Scopus, and Web of Science for articles published between January 2010 and June 2025. Overall, this review underscores the growing potential of multifunctional dental materials that integrate bioactivity with antimicrobial performance, offering promising directions for the development of next-generation restorative and implant materials.

## 1. Introduction

The widespread use of implants, such as catheters, prosthetics, and various medical devices, has significantly transformed the field of medicine in recent years. Modern medical implants gained widespread use in the mid-20th century, and millions of different types are implanted in North America each year. Consequently, dental implants have been widely utilized as a means of supporting prosthetic teeth, with a long and complex history of development. According to data from the American Association of Oral and Maxillofacial Surgeons, approximately 69% of adults between the ages of 35 and 44 have lost at least one permanent tooth due to factors such as trauma, periodontal disease, failed endodontic treatment, or dental caries [1]. Additionally, by the age of 74, approximately 26% of individuals experience complete edentulism. Annually, the placement of dental implants has become increasingly common, with an estimated 100,000 to 300,000 implants inserted [2].

In recent years, research on dental implant designs, biomaterials, and surgical techniques has expanded significantly and is anticipated to continue growing. This trend is driven by the rapid expansion of the global dental implant market and the increasing demand for esthetic and restorative dental treatments [3].

However, these devices carry a high risk of infection, making implant-related infections some of the most common and serious complications associated with biomaterials. In the United States, infections related to medical devices represent about 26% of healthcare-associated infections [4,5]. Orthopedic and dental implants, which are intended to remain inside the body, pose particular challenges, as infections can lead to prosthetic failure, often necessitating implant replacement and resulting in chronic or recurrent issues [6]. Diagnosing infections in these implants is complex and requires identifying the specific pathogen and assessing its drug susceptibility. Treatment is further complicated by challenges such as antimicrobial resistance and persistent infections [7].

**Figure 1:**
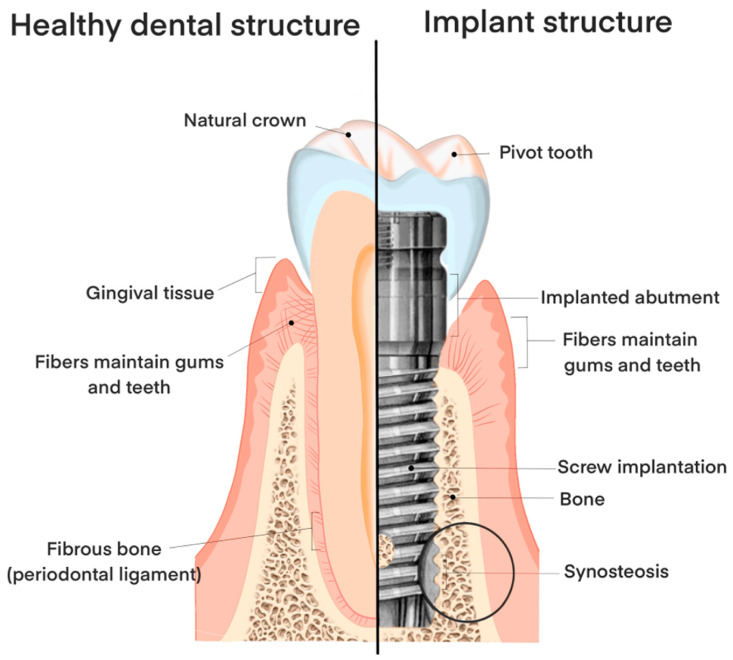
Typical structure of a dental implant [8].

Dental implants consist of the implant body and its supporting components, the abutment and crown, functioning as an artificial tooth root. These are fabricated from biocompatible synthetic materials and surgically placed into the jawbone at the site of a missing tooth. Implants serve as an effective restorative option, offering superior stability and strength compared to conventional dental restorations. Moreover, they enhance esthetics, provide greater comfort than traditional dentures, preserve adjacent natural teeth, prevent alveolar bone resorption, and help maintain facial structure. Overall, dental implants closely replicate the form, function, and appearance of natural teeth, as illustrated in Figure 1 through a structural comparison between a healthy tooth and an implant.

A comprehensive literature search was conducted within PubMed, Scopus, and Web of Science for articles published between January 2010 and June 2025, using the keywords “classifications of antimicrobial materials”, “classification of biomaterials”, and “dental materials.” Only peer-reviewed articles written in English were included. Editorials, conference abstracts, and duplicate studies were excluded. A flow diagram of the systematic review is found in the Supplementary Data.

## 2. Challenges Associated with Dental Implants

Dental implants should be long-lasting and durable, but they can sometimes fail over time. Ensuring their longevity is essential for optimal performance and patient satisfaction. Many implants have seen substantial advancements; however, there are still certain issues and obstacles linked with them. These difficulties lead to failure, shorten life, and may even endanger human life. Dental and orthopedic implants are widely used to restore function and esthetics. However, they can be associated with various challenges, as summarized below.

### 2.1. Post Operative Infection

After dental implant insertion, a large sum of patients experienced postoperative infections. These are treated with prescribed antibiotics, but most are resistant to antibiotics, and 15 Canadians per day were estimated to have lost their lives to antimicrobial-resistant infections [9,10]. The resistance rate will reach 40% by 2050; 13,700 individuals will die per year from resistant bacterial infections [9]. According to estimates, 26 percent of bacterial infections in Canada are already resistant to the first prescription medications generally used to treat them [10]. Antibiotic failure necessitated surgical retreatment for 90% of the infected patients, and 65% of the impacted implants were removed [11].

Surgical procedures and the presence of foreign bodies further exacerbate the risk of infection by causing tissue damage, activating immune responses, and triggering the production of inflammatory mediators, which are intensified by bacterial toxins and activity [12]. Certain bacteria, such as *Staphylococcus epidermidis*, which are typically non-virulent, can evade immune defenses and antibiotic treatments [12]. This has driven the development of alternative strategies, including infection-resistant materials designed to act as antimicrobial drug-delivery systems. These systems enable the localized, sustained release of antimicrobial agents around the implant site, avoiding systemic side effects and achieving drug concentrations that are far higher than conventional systemic treatments.

The bioengineering of hybrid implant materials is advancing rapidly, focusing on optimizing device performance while minimizing inflammatory reactions and cellular disorganization at the interface. These innovative materials, capable of slowly releasing antimicrobial agents, hold promise for reducing implant-related infections in the future.

### 2.2. Implant Rejection

**Figure 2:**
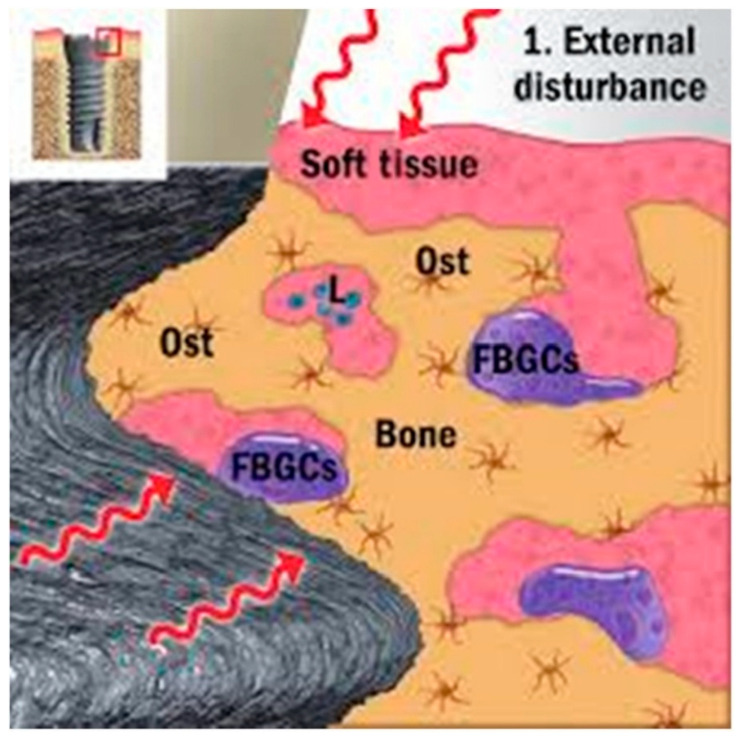
Diagram showing the “foreign body equilibrium” disruption theory of peri-implant bone loss. This notion views the osseointegrated implant as “encapsulated in bone,” indicating a persistent inflammatory state with “foreign body giant cells” (FBGCs). In response to a variety of external stimuli, these foreign body giant cells are activated and are responsible for the peri-implant bone loss. L means “lymphocyte”; Ost means “osteocyte”. Diagram adapted with permission from [13].

The body may react adversely to an implant, viewing it as a foreign object. There is evidence of a persistent foreign body reaction to osseointegrated dental implants and their possible role in crestal bone loss, characteristic of peri-implantitis [13]. The release of implant-related materials, including titanium particles and corrosion by-products, into the surrounding tissue plays a significant role in the onset and advancement of peri-implantitis, leading to rejection [14]. Rejections may be long term responses, further fusing into foreign body giant cells (FBGC), while bone cells make and remodel hydroxyl apatite. The above sequence results in osseointegration (shown in Figure 2). The lifespan of an implant depends on maintaining a balance with the surrounding tissues. If this equilibrium is disrupted, it can lead to reduced functionality through a process in which macrophages become activated and form FBGCs in larger numbers. This can trigger bone resorption, as cells like osteoclasts, and possibly even macrophages, break down more bone than osteoblasts can rebuild. Additionally, mucosal seals may rupture through complex mechanisms. Secondary infections can further complicate the situation, potentially leading to implant failure [14]. Although true implant rejection is rare, hyperallergic reactions to materials such as metals can cause inflammation and discomfort.

### 2.3. Allergic Reactions

Specific materials, primarily metals used in implants, may induce allergic responses in sensitive individuals, resulting in pain, edema, or inflammation. Metals such as nickel, chromium, and cobalt, as well as bone cement constituents such acrylates and gentamicin, may induce intolerant responses to implants [15]. Metal allergies in dentistry are common, with nickel allergies being up to 20%, making them a leading cause of allergic dermatitis. In industrialized countries, it ranks as the top allergen [16]. Among orthodontic patients, 30% are allergic to nickel, copper, and chromium. Though rare, allergies to other metals like mercury, gold, platinum, palladium, silver, and cobalt can also occur [16]. Eczemas were mainly observed after osteosynthesis in sensitive patients implanted with material made from nickel, chromium, or cobalt [15]. Type IV (delayed) allergic hypersensitivity reaction may cause fistula formation, eczema, and itching of the skin or mucosa due to reactions from restorative composites of fissure sealants, bonding agents, and orthodontic and crown and bridge resins made from polymerized poly(methyl methacrylate) (PMMA) “pellets” with additives dibenzoyl peroxide, N,N-Dimethyl-p-toluidine, or 2-[4-(Dimethylamino)phenyl]ethanol [15].

### 2.4. Peri-Implantitis

**Figure 3:**
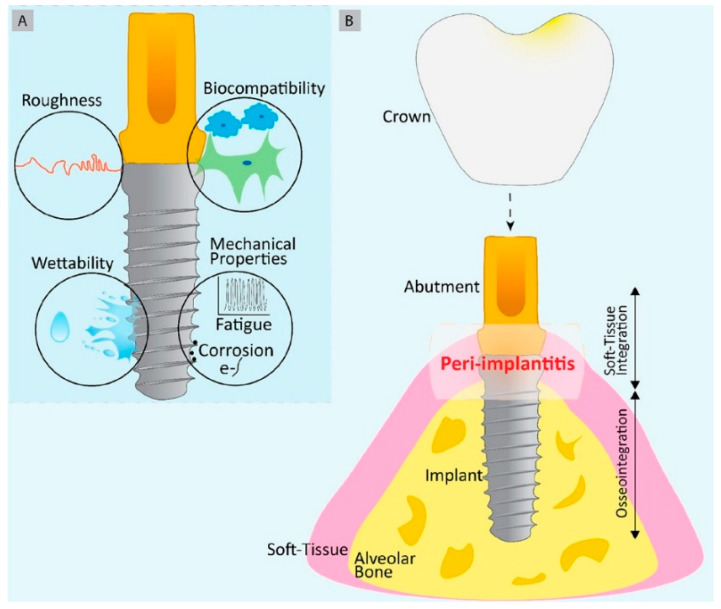
(**A**) Dental implant design. (**B**) Components with major regions of interest involved in peri-implantitis [19]. Copyright © 2022, American Chemical Society).

Especially in dental implants, peri-implantitis involves inflammation around the surrounding tissues and can lead to bone loss. Peri-implant disease occurs when the soft tissue around a dental implant becomes infected and begins to break down. This may result in pain, swelling, difficulty in biting and chewing, and, if untreated, potential failure [17]. Treatment for peri-implantitis often entails a mix of mechanical debridement and antibiotic treatment [18,19]. Ongoing research involves modification of dental implants that could reduce peri-implantitis, as shown in Figure 3.

### 2.5. Implant Failure

Mechanical issues, such as wear and tear, stress fractures, or loosening over time, can lead to implant failure [20]. This is common in weight-bearing implants: especially dental, where the implant might wear out due to stress [21]. To minimize failure, implants are tested on biomechanical evaluation, which includes tests to assess their mechanical properties, such as compression, tension, and torsion [22].

Occlusal stress due to the mechanical load transmitted to dental implants during mastication and parafunctional activity. Excessive or uneven occlusal forces can cause microstrain in peri-implant bone, leading to marginal bone loss, fatigue, or implant fracture. Proper occlusal design, balanced load distribution, use of resilient prosthetic materials, and management of bruxism are essential to mitigate these effects. Effective control of occlusal stress through biomechanical planning significantly enhances the long-term stability and survival of implants [23,24].

### 2.6. Bone Loss or Resorption

Dental implants may lead to tooth loss around the implant site due to a lack of bone stimulation or other factors like infection, which can compromise the implant’s stability. The literature on commercially available implant systems reports 6% bone loss in the first year, 10% in the first 10 years, and 12% in the 15 years after surgery [25]. So, when designing such implants that may be prone to bone loss, we must consider biocompatible materials facilitating osteointegration, thread designs reducing bone to implant contacts, and the appropriate geometry, length and size [26].

### 2.7. Esthetic Issue

In cases of cosmetic implants, there may be issues with symmetry, shape, or placement, which can impact patient satisfaction and may require revision surgery.

## 3. Implant-Related Dental Infections

To conclude, it can be stated that infection due to dental implants may be a major challenge, as it increased the rate of implant failure. The osseointegration process may be hampered by microbial infections, which may ultimately result in the implant’s removal [27]. Peri-implantitis and peri-implant mucositis are the primary infectious complications that cause implant loss. They are brought on by the patient’s immune system, which causes an inflammatory process in the bone and mucosa surrounding the implant, both of which are linked to the organized microorganisms in biofilm [28]. It is believed that peri-implant disorders occur at a rate of about 30%, with smokers having a greater prevalence [29].

With around 700 species of bacteria, fungi, viruses, and protozoa that interact with one another either antagonistically, cooperatively, or even as signaling agents, the oral microbiome is the second biggest in the human body. A highly structured community known as biofilm is formed by these oral bacteria adhering to the biotic or abiotic substrate as well as to one another [30]. In their many settings, microorganisms can appear as communities, which is their preferred form, or in their free form, known as planktonic microorganisms. Biofilm is the collective term for the group of microorganisms affixed to a surface.

### 3.1. Microbiota of Oral Cavity and Dental Implants

**Oral Microbiota** [31]: The oral cavity represents a complex and dynamic environment composed of several distinct microhabitats, including the teeth, buccal mucosa, hard and soft palates, and tongue. The oral microbiota consists of a wide array of microorganisms, including bacteria, fungi, and viruses. Among these, bacteria constitute the predominant microbial population, primarily belonging to the phyla *Firmicutes*, *Bacteroidetes*, *Proteobacteria*, and *Actinobacteria*. In contrast to the gut microbiota, the composition of oral bacterial communities exhibits relative stability and is less influenced by external factors such as diet and environment. Interestingly, the oral microbiota of healthy individuals tends to be highly conserved across different geographic and ethnic populations.

The oral mycobiome includes approximately 85 fungal species, with *Candida* spp., particularly *Candida albicans*, being the most frequently detected and clinically significant. Under the conditions of microbial homeostasis, Candida species are generally commensal; however, when dysbiosis occurs, they can shift to an opportunistic pathogenic role. Notably, Candida can co-aggregate with *Streptococcus* spp. to form pathogenic biofilms that contribute to oral diseases such as candidiasis and caries.

Among the bacterial species, several are of particular clinical relevance. Streptococcus mutans, a Gram-positive facultative anaerobe, is a key colonizer of the dental biofilm and a primary etiological agent of dental caries, which is the most prevalent chronic disease affecting the hard tissues of the teeth. *Porphyromonas gingivalis*, a Gram-negative anaerobic and asaccharolytic bacterium, is a major periodontal pathogen implicated in the progression of periodontitis. Chronic colonization by *P. gingivalis* can lead to destruction of the periodontal ligament and eventual tooth loss. Members of the genus Lactobacillus—including *Lactobacillus acidophilus* and related species—are lactic acid-producing bacteria that, while often considered beneficial in gastrointestinal contexts (e.g., as probiotics), are also associated with caries development due to their acidogenic and aciduric properties. Other notable genera present in the oral cavity include Staphylococcus, which may play a role in opportunistic infections, and Prevotella, Dialister, and Filifactor, which have been implicated in both dental caries and periodontal disease through recent metagenomic analyses.

**Implant Microbiota**: The bacterial species identified included *Staphylococcus epidermidis*, *Eubacterium* spp., *Corynebacterium* spp., and *Streptococcus viridans*. *Staphylococcus epidermidis* was isolated from most patients and exhibited resistance to penicillin, a commonly preferred antibiotic among clinicians [32]. A summary of the most common bacteria isolated from dental implants that have failed due to infection is represented in Table 1.

### 3.2. Biofilms on Implants

Biofilms are the predominant form of microbial life, consisting of a biologically active matrix of cells and extracellular substances attached to implant surfaces. They are composed of numerous bacteria embedded in an organic polymeric material. The extra-cellular polysaccharides (EPS) are a slimy and insoluble fluid produced by bacterial cells, surrounded by millions of neighboring microbes within a well-organized, structured matrix. This EPS matrix has key properties that are necessary for the bacteria within biofilms [34]. First, EPS facilitates the distribution of nutrients essential for cell growth [34]. Second, its diverse composition of charged polysaccharide groups efficiently traps the external nutrients necessary for cell survival and proliferation [34]. Third, the EPS matrix offers enhanced protection to encapsulated cells from environmental stresses compared to free-floating, planktonic bacteria [35]. Biofilms also provide advantages such as resistance to antibiotics [36], biocides [37], and harsh environments [38].

**Figure 4:**
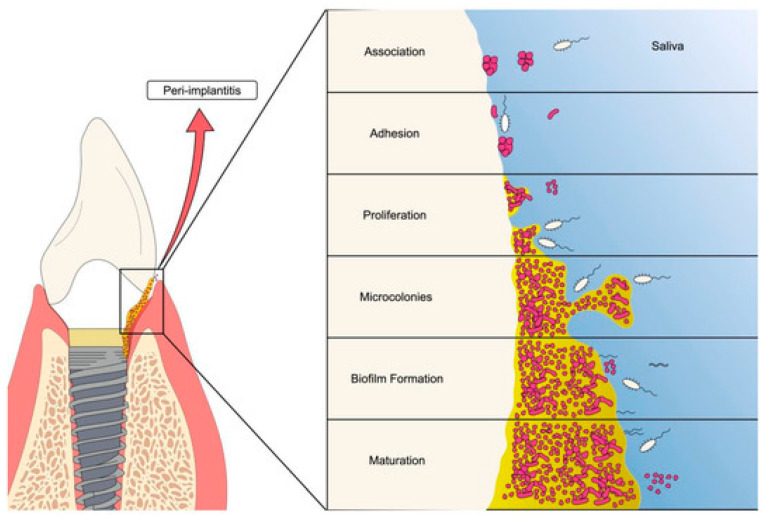
Process of oral biofilm formation on dental implants [46].

The first stage of biofilm involves the quick adhesion of microbes to the surface of the medical devices and proliferation of cells (Figure 1, Figure 2, Figure 3 and Figure 4). The attachment of bacteria is initially dependent on polarity, London–van der Waals forces and hydrophobic interactions [39]. There are various bacteria that are adhered to the protein surface, contributing to the initial adhesion. The first stage involves the capsular polysaccharide—adhesion (PS/A) to enable attachment and slime production [40].

The second stage involves the formation of the colonization of bacteria. Bacterial cell multiplication and intercellular bonding occur once the microorganisms are secured to the implants’ surface. Polysaccharide intercellular adhesion (PIA) is a polysaccharide antigen that promotes intercellular adhesion and biofilm formation in Staphylococci [41]. Colonies are enclosed by an EPS, gaining protection inside the EPS, and form larger macro colonies [42]. The cell proliferation and maturation processes further continue (as shown in Figure 1, Figure 2, Figure 3 and Figure 4: steps 3 and step 4). In the last stage, the biofilm reaches a critical mass and due to a depletion in nutrients, planktonic bacteria disperse from the surface [43]. The dispersed bacteria leaves the macro-colony and moves into the bloodstream, spreading infection elsewhere. The manner of dispersal varies between species, influencing their morphological characteristics. *S. aureus* disperses and recolonizes a surface after approximately 6 h [44], *V. parahaemolyticus* after 4 h, and *V. harveyi* only recolonizes after 2 h [45].

Quorum sensing has been shown to regulate biofilm differentiation and, in some Gram-negative bacteria, can result in the destruction of leukocytes. The EPS production, combined with the altered properties of biofilm-associated bacteria, provides protection, making it challenging for the immune system and antibiotics to eliminate these embedded cells once on a surface. As a result, biofilm infections often become chronic. Additionally, biofilm bacteria typically trigger a weaker inflammatory response compared to planktonic bacteria, complicating the treatment of such infections.

## 4. Dental Antimicrobial Approaches

Like periodontal diseases, peri-implant diseases are linked to the accumulation of dental plaque on implants. Various unconventional methods have been explored for plaque removal from infected implants; however, none can fully and permanently eliminate bacterial invasion. Fortunately, the ongoing advancement of antibacterial implant materials offers a promising solution. This review outlines the development and evaluation of different antibacterial strategies for dental implant materials aimed at preventing peri-implantitis. By emphasizing the benefits and limitations of these approaches, we hope to contribute to the continuous improvement of oral implant materials. Many of the solutions to the challenges discussed in Section 2.2 may be addressed through proactive measures to prevent bacterial colonization by engineering the implant surface to confer antimicrobial properties. This represents a line of development pursued in recent years.

**Figure 5:**
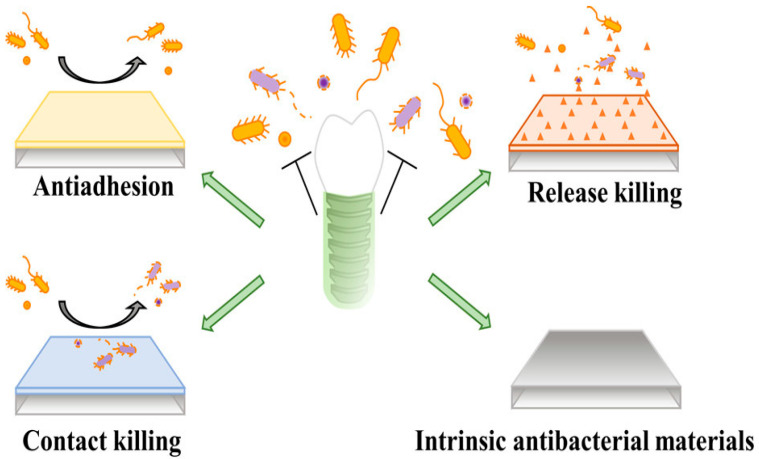
Types of antimicrobial coatings categorized into contact-killing, releasing, antifouling, smart, and composite types, based on their mechanism of action. Each type aims to prevent microbial adhesion or eliminate pathogens, enhancing the durability and infection resistance of dental and biomedical implants [47]. (Reused with permission. Copyright © 2021. Published by the American Chemical Society. Licensed under CC-BY-NC-ND 4.0).

**Figure 6:**
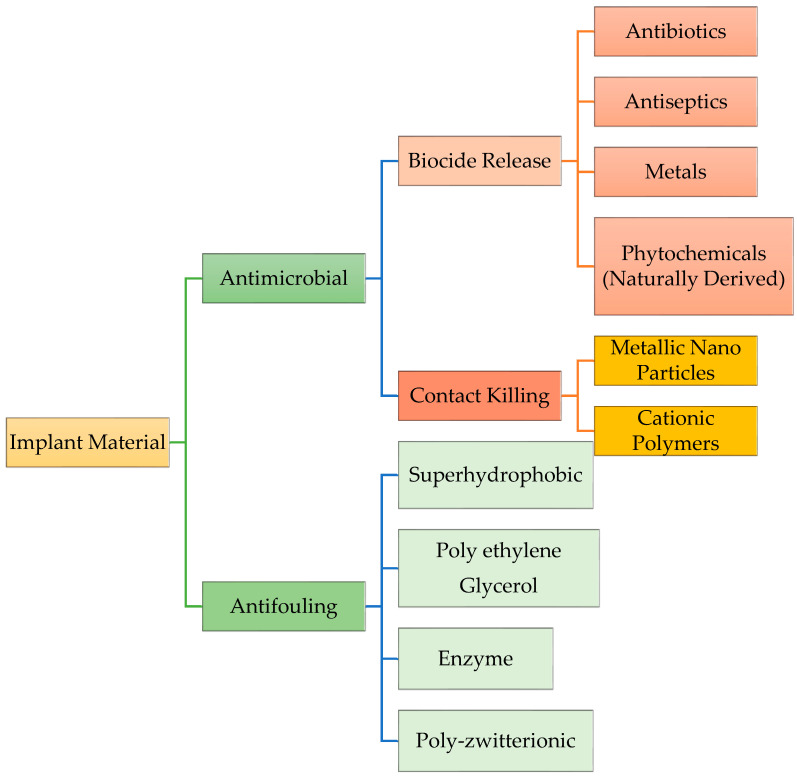
Classification of anti-infective biomaterials: release behavior (contact-killing vs. release-based), type of agent (metallic, organic, polymeric, or natural), or anti-adhesive (antifouling).

Figure 5 outlines the methods for antimicrobial coating types. One of the approaches used to prevent infection is to develop coatings for implants that microbes find difficult to colonize in the first place, known as antimicrobial surfaces. Antimicrobial material prevents the adherence of bacteria by repelling properties and prevents the biofilm formation as shown in Figure 6, following their sub-division of preventive strategies. Section 4.1, Section 4.2, Section 4.3 and Section 4.4 outline the primary types of antimicrobial materials used in dentistry.

### 4.1. AMPs

Antimicrobial peptides (AMPs) are short peptides with broad-spectrum antibacterial properties that interact with bacterial cell membranes, leading to bacterial death [48]. Some common usages of AMPs in dentistry are listed in Table 2. Unlike antibiotics, AMPs have a different bactericidal mechanism, making them less likely to cause drug resistance. Studies have shown that AMP-functionalized surfaces exhibit strong antibacterial effects with low cytotoxicity. For instance, AMPs immobilized on titanium can inhibit bacterial adhesion while promoting osteoblast activity [49]. To further optimize AMP-functionalized surfaces, factors such as AMP properties, spacer selection, and AMP density should be considered. AMP-functionalized surfaces in dental materials, particularly on dental implants, generally show low cytotoxicity towards human cells while exhibiting strong antibacterial effects. This balance is a key advantage of using antimicrobial peptides (AMPs) compared to some conventional antibiotics or metal-releasing coatings, which can have higher toxicity concerns [50].

There are two main methods for immobilizing AMPs on implant surfaces: physical adsorption and covalent immobilization [52]. While physical adsorption is simple, it only provides a short-term antibacterial effect, due to limited AMP attachment. In contrast, covalent immobilization ensures long-term stability and enhanced bioactivity by properly orienting AMPs for effective bacterial interaction. Research has demonstrated that controlling AMPs’ orientation significantly improves their antibacterial efficiency [53].

### 4.2. Metal-Releasing Coatings

Antibacterial metals and their alloys display an increased ability to prevent bacterial adhesion, development, and cell proliferation, through element alloying and exposure to heat. In recent years, Cu- and Ag-containing antibacterial metal alloys have been shown to be effective against a wide range of germs, including antibacterial titanium and its alloys. The components that make up the alloy are mostly Ag and Cu, which have been shown to have wide-ranging antibacterial effects. Table 2 and Table 3 summarizes each metal used: their features, toxicity, and antimicrobial analysis.

### 4.3. Phytochemicals Used in Dental Materials (Phytodentistry)

Plant-derived chemicals may improve dental biomaterials’ physicochemical qualities and aid oral health. To improve dental biomaterial performance, plant polysaccharides, proteins, and bioactive phytochemical-rich extracts are used. Despite strong evidence that plant-derived compounds increase material–tissue and cell interactions, research on potential novel dental biomaterials is scarce. Only a few studies have explored plant extract-based titanium implant coatings and periodontal regenerative materials, highlighting the need for further investigation in this promising area. These extracts and compounds are difficult to obtain, needing long and complex protocols of extraction, chemical characterization, and isolation, often with a low yield. In some cases, isolating a single compound in significant amounts remains a challenge. Table 4 highlights examples of phytochemicals responsible for antimicrobial properties and their applications:

### 4.4. Quaternary Ammonium Compounds

Quaternary ammonium salts (QAS) are widely employed in the food industry, textiles, surface compounds, and water purification, due to their broad-spectrum antimicrobial properties and low toxicity. Their antibacterial properties are derived from their capacity to bind to bacterial membranes, which results in bacterial lysis [84]. When negatively charged bacterial cells meet the positively charged quaternary amine group (N+), the electrical balance is disrupted, causing the bacteria to rupture under osmotic pressure [85]. Long-chain cationic polymers may also enter bacterial cells, puncturing their membranes in the same way as a needle does when bursting an air sac.

**Figure 7:**
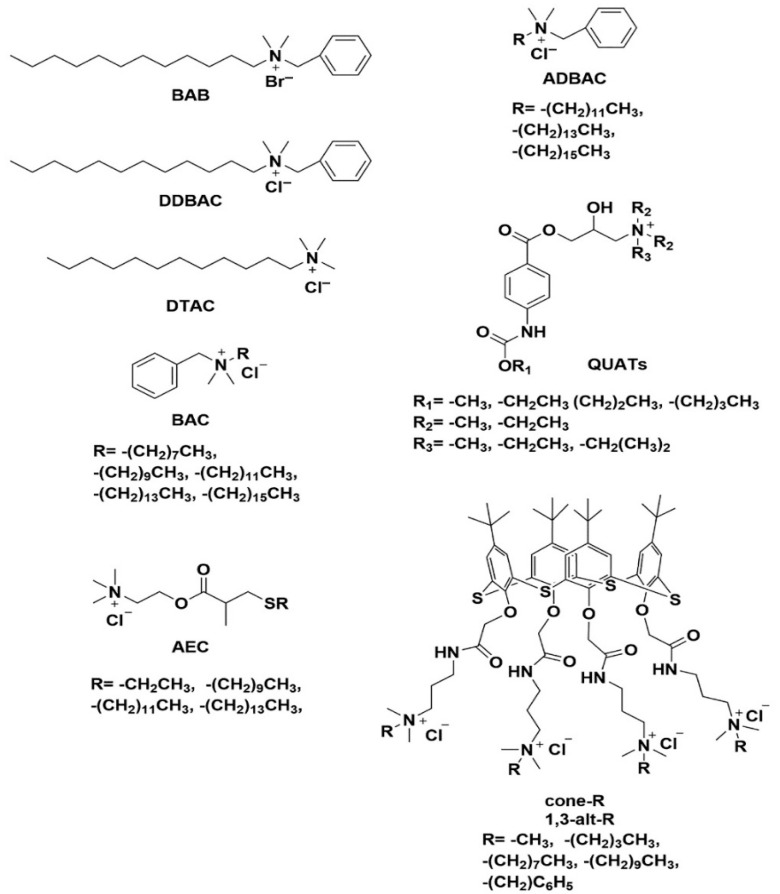
Chemical structures of different QASs. (Used with permission from [92]).

Research on the synthesis of novel quaternary ammonium monomers aims to identify compounds with strong antibacterial effects, low cytotoxicity, affordability, ease of production, and minimal impact on mechanical properties [86]. For over three decades, antimicrobial QAS monomers have been included into composite materials to inhibit plaque accumulation and secondary caries. The notion of “immobilized bactericide” was established in dentistry to guarantee sustained antibacterial efficacy while maintaining mechanical integrity. In 1994, the incorporation of a quaternary ammonium monomer into dental composites was introduced [87,88]. Since then, various QAMs have been synthesized and incorporated into materials like glass ionomer cement (GIC), etching-bonding systems, and resin composites to enhance their antibacterial properties [89]. This review provides an overview of previous studies on dental materials incorporating QASs, serving as a foundation for the subsequent chapters of this thesis, which focus on the synthesis and antimicrobial analysis of QASs in dental implants. A number of QACs and their minimum inhibitory concentrations are summarized in Table 5 and their toxicity comparisons were elaborately discussed. Some common QACs structures are shown in Figure 7. 

**Single Chain QAS:** Studies on composite materials containing antibacterial components released over time have been reported by a number of researchers [90]. Reports of such materials were evaluated by Chen and colleagues [90]. In their review, they classified antibacterial chemicals into three groups: (1) leachable compounds like chlorhexidine and benzalkonium chloride, (2) polymerizable monomers like quaternary ammonium (QA) methacrylates, and (3) filler particles like nano silver. Even though many antibacterial compounds were investigated between 2012 and 2017, only four agents—benzalkonium chloride, chlorhexidine, glutaraldehyde, and 12-methacryloyloxydodecylpyridinium bromide—were included in commercial goods.

FDA has approved the human consumption and use of many quaternary ammonium compounds, which may be safely used given following conditions [91]:

- The additive contains the following compounds: *n*-dodecyl dimethyl benzyl ammonium chloride (CAS Reg. No. 139-07-1); *n*-dodecyl dimethyl ethylbenzyl ammonium chloride (CAS Reg. No. 27479-28-3); *n*-hexadecyl dimethyl benzyl ammonium chloride (CAS Reg. No. 122-18-9); *n*-octadecyl dimethyl benzyl ammonium chloride (CAS Reg. No. 122-19-0); *n*-tetradecyl dimethyl benzyl ammonium chloride (CAS Reg. No. 139-08-2); *n*-tetradecyl dimethyl ethylbenzyl ammonium chloride (CAS Reg. No. 27479-29-4).
- The composition meets the following specifications: pH (5 percent active solution) 7.0–8.0; total amines, maximum 1 percent as combined free amines and amine hydrochlorides.
- The compound is used as an antimicrobial agent, as defined [91] orally in food.

**Table 5:** Different QASs, target bacteria/fungus and their minimum inhibitory concentration (MIC).

Name of QAS	Target Bacterial Strain	Human Cell Toxicity	Reference	
**Alkyl Dimethyl benzyl Ammonium Chloride (ADBAC)**	*S. aureus*; MIC: 0.6 μg mL^−1^	In chronic trials with beagles, mice, and rats, repeated dosage oral toxicity studies found no harmful effects at 10–93.1 mg/kg-day for DDAC and 3.7–188 mg/kg-day for ADBAC (C > 12). At modest adverse impact levels, DDAC and ADBAC (C > 12) consistently cause decreased food intake, average body weight, body weight growth, and localized discomfort.	[93,94]	
**Dodecyl dimethyl benzyl ammonium chloride (DDBAC)**	*Listeria monocytogenes*; *E. coli*; *S. aureus*	Cell viability (NIH-3T3 assays) was 39.7% within 24 hrs incubation at dose of 500 μg/mL, respectively.	[95]	
**P-tert-butylthiacalix [4]arene (1,3-alt-R)**	*S. aureus*, *B. subtills*, *E.coli*, *P. aeruginosa*	Toxicity tests on human skin cells showed less toxicity as compared to ref. drugs.	[96]	
**Ammonium-esterified acrylate (AEC)**	*S. aureus*; MIC: 3 ppm, *E.coli*; MIC: 31 ppm, *P. aeruginosa;* MIC: 250 ppm, *Candida albicans*, *Aspergillus niger*; *Klebsiella pneumoniae*; *Acinetobacter baummanii*	_	[97]	
**Didecyl dimethylammonium chloride (DDAC)**	*S. aureus;* MIC: 1.63 µM, *E.coli*; MIC: 15. 63 µM, *P. aeruginosa*; MIC: 500 µM; *K. pneumoniae*; MIC: 11 µM, *Enterococcus* sp.; MIC: 3 µM.	Cell viability assays confirm a trend of a higher cytotoxicity in correlation with an increasing carbon chain length of the compounds. DDACs prove more effective as surface disinfectants than antiseptics, due to their hazardous potential and wide range of selectivity for bacteria.	[98]	
***N*** **,*N*-dialkyl-*N*-(2-hydroxyethyl)-*N*-methylammonium salts (NDMAC)**	*S. aureus*; MIC: 0.9 µM, *E.coli*; MIC: 7.8 µM, *P. aeruginosa*; MIC: 500 µM			
***N*-[*N*′(3-gluconamide)propyl-*N*′-alkyl]propyl-*N*,*N*-dimethyl-*N*-alkyl ammonium bromide (CDDGPB)**	*S. aureus;* MIC: 150 ppm, *E.coli*; MIC: 150 ppm,	The mortality of mice test group was the highest, with an LD_50_ of mice larger than 100 mg/kg, indicating that the surfactant has medium toxicity. The mortality of mice in the C_10_DDGPB test group was significantly lower than that in the C_12_DDGPB test group. No obvious blackening or body stiffness was observed in any of the tested animals during the 14 day observation period.	[99]	

**QAS with multiple chain lengths:** Increasing the alkyl chain length (CL) enhanced hydrophobicity, potentially improving the capacity to traverse the hydrophobic bacterial membrane. Cationic polymers with longer chain lengths may more effectively penetrate bacterial cells and disrupt membranes [100]. As a consequence, multiple studies sought to synthesize QAS with different chain lengths before testing its anti-caries potential in various dental materials. A recent study on glass ionomers found that increasing the chain length significantly improved antibacterial activity [101]. A series of QAS molecules with different chain lengths were synthesized in a separate study, including dimethylaminopropyl methacrylate (DMAPM, CL = 3), dimethylaminohexyl methacrylate (DMAHM, CL = 6), dimethylaminononyl methacrylate (DMANM, CL = 9), dimethylaminododecyl methacrylate (DMADDM, CL = 12), dimethylaminohexadecyl methacrylate (DMAHDM, CL = 16), and dimethylaminooctadecyl methacrylate (DMAODM, CL = 18). Two antibacterial monomers, DMADDM with a chain length of 12 and DMAHM with a chain length of 6, were selected for further investigation. MIC, minimum bactericidal concentration, and ADT assays, DMAHM and DMADDM, demonstrated significantly stronger antibacterial activity compared to the previous QADM. Furthermore, DMAHM, with a chain length of six, demonstrated significantly lower effectiveness compared to DMADDM, which possessed a chain length of twelve [102]. Owing to their strong antibacterial characteristics, two of these monomers, DMADDM with a carbon chain length of 12 and DMAHDM with a carbon chain length of 16, were thoroughly investigated as anti-caries agents in diverse dental materials.

As anti-caries agents, DMADDM was added to orthodontic cement, resin composite, and adhesives [103].

Additionally, a Taqman real-time polymerase chain reaction was used to examine the proportional change in multispecies biofilms with varying mass fractions of DMADDM. The results indicated a consistent decrease in the ratio of Streptococcus gordonii throughout time, but the ratio of Streptococcus mutans in biofilms increased in adhesives devoid of DMADDM. Nevertheless, the proportion of Streptococcus mutans markedly decreased, whereas the proportion of Streptococcus gordonii regularly rose in the adhesives [104]. According to reports, Streptococcus gordonii is linked to healthy enamel and is an early colonizer of the dental plaque biofilm [105]. As a result, following DMADDM control, the biofilm has a propensity for healthier growth. DMADDM-containing adhesives inhibit MMPs, preventing hybrid layer degradation and enhancing dentin–resin bond durability [106].

Chen et al. developed a bonding agent by integrating DMADDM and amorphous calcium phosphate (NACP) nanoparticles into primer and adhesive, which has both antibacterial and remineralizing capabilities. NACP in the adhesive released Ca and P ions for remineralization and caries inhibition, whereas DMADDM in the bonding agent had a potent antibacterial activity [107]. At one day and one month, this was accomplished without sacrificing the dentin bond strength. Bonding agents containing DMADDM and NACP were water-aged for six months in another long-term experiment. The results indicated that the innovative anti-caries adhesives exhibited robust and enduring antibacterial capabilities, together with a markedly superior bond strength compared to a commercial control after six months of water aging [108].

A separate in vitro study investigated the impact of chain length variations in QAS on cytotoxicity. The cytotoxicity of QAS against fibroblasts and odontoblasts, with chain lengths between three and sixteen, was comparable to that of commercial controls [109]. A methyl thiazolyltetrazolium test and a live/dead viability assay were used to evaluate the cytotoxicity of DMADDM on human gingival fibroblasts (HGF). The results indicated that BisGMA, a prevalent component in commercial products, had much more cytotoxicity than DMADDM [110]. A rat tooth model was used to study pulpal inflammation, tertiary dentin formation, and restoratives such NACP and DMADDM. DMADDM showed no effect on pulpal inflammation compared to commercial glue and glass-filled composites [111].

In order to create new antibacterial dental materials, DMAHDM with a chain length of 16 was also added to resin composites and adhesives. Microcosm biofilm CFU may be reduced by 4 log, using adhesives containing 10% DMAHDM [112]. In another in vitro study, primers and adhesives received DMAHDM at 0%, 2.5%, 5%, 7.5%, and 10% mass fractions. As the resin’s DMAHDM mass fraction grew, bacteria’s early attachment coverage decreased. Since DMAHDM does not improve dentin bond strength [113], DMAHDM was administered for dental caries in conjunction with other efficacious therapies, such as NACP and 2-methacryloyloxyethyl phosphorylcholine (MPC), to attain dual or triple benefits in caries prevention [114].

## 5. Bioactive Dental Materials

### 5.1. Properties of Biomaterials

Biomaterials are materials designed to interact safely and effectively with the human body to restore, repair, or improve biological tissues. They play a crucial role in the creation of implants, devices, and systems that support healing and enhance the quality of life across various medical and biological applications. The main properties of biomaterials have to be as follows:

**Biocompatible:** To ensure that the body does not reject a material or trigger an inflammatory response. Some biomaterials can even regulate biological reactions with precision, influencing processes such as cell adhesion, cell growth, and the formation of blood vessels [115].

**Mechanical properties:** Dental implants, for instance, need to be robust and have enough load-bearing capacity to withstand the mechanical stresses of bone [116].

**Degradability:** To naturally break down over time, which can be beneficial in certain cases, allowing for the gradual replacement of the biomaterial by surrounding the biological tissue. This is especially important in situations where the material needs to integrate permanently with the body, such as in temporary applications [117].

### 5.2. Metallic Substrates

Titanium (Ti) and its alloys are widely used to make orthopedic and dental implants because of its corrosion resistance, biocompatibility, low elastic modulus, and high fatigue strength [117]. Among these, commercially pure alpha titanium (CpTi) and the alpha-beta Ti-6Al-4V alloy are the most commonly employed materials for such biomedical applications. Due to its good mechanical performance and inexpensive cost, stainless steel, especially AISI 316L (316L SS), is still commonly utilized; however, metal ions from corrosion and wear remain a worry [118]. In comparison to stainless steel and cobalt–chromium alloys, titanium and its alloys exhibit superior mechanical and biological characteristics [61,119].

In addition to non-resorbable metallic materials, magnesium (Mg) and its alloys are being explored for orthopedic applications due to their biodegradability and potential for temporary support [62]. However, their rapid degradation rate renders them unsuitable for dental implant use, where long-term structural integrity is essential [120].

Osseointegration is widely recognized as a critical determinant for the long-term success of biomedical implants. To enhance early implant stability and reduce the time required for effective osseointegration, various surface modification strategies have been investigated [121]. Surface roughness, in particular, plays a crucial role in mediating bone–implant interactions, as it affects cellular responses such as adhesion, proliferation, and differentiation. However, achieving the optimal surface topography is complex; excessively rough surfaces may promote bacterial colonization and peri-implantitis, while surfaces that are too smooth may impair osseointegration [61]. As a result, several investigations have concentrated on creating altered implant surfaces using physical and chemical methods, including sandblasting, acid cleaning, mixed blasting and etching, electrochemical oxidation, and treatment with lasers [121,122].

**Drawbacks of uncoated metal substrates are as follows:**

**Poor biocompatibility**: Some metals may not integrate well with the surrounding bone and tissue, leading to implant failure or rejection. Coatings like titanium oxide or hydroxyapatite improve biocompatibility and osseointegration. Osseointegration has been considered as a key factor for the long-term success of biomedical implants. In order to obtain improved osseointegration and to shorten the time for osseointegration, enhancing implant stability in the early phases, several implant surface modifications have been explored [121].

**Poor osseointegration**: Uncoated metals may not bond effectively with bone, leading to implant loosening or failure. Coatings enhance osseointegration by promoting bone growth around the implant. For instance, the surface roughness has been demonstrated to affect the bone–implant interactions [61]; numerous studies have endeavored to create changed surfaces through various physical and chemical methods, including as sandblasting, acid etching, a combination of blasting and etching, electrochemical oxidation, and laser treatments. Coating materials may also be used to enhance the surfaces of implants, hence improving the performance of metallic implants. Surface characteristics of materials significantly influence chemical and biological interactions with adjacent bone tissue, while mechanical qualities are mostly dictated by the implant’s mass [61].

In contrast, poor mechanical properties of monolithic bioceramics and bioactive glasses limit their use in load-bearing applications. As a consequence, the materials of choice still remain metallic alloys, whose biological properties can be improved by the means of coatings (e.g., bioactivity, reduction in corrosion and toxic ion release) [123,124,125].

### 5.3. Bioactive Glass

**Figure 8:**
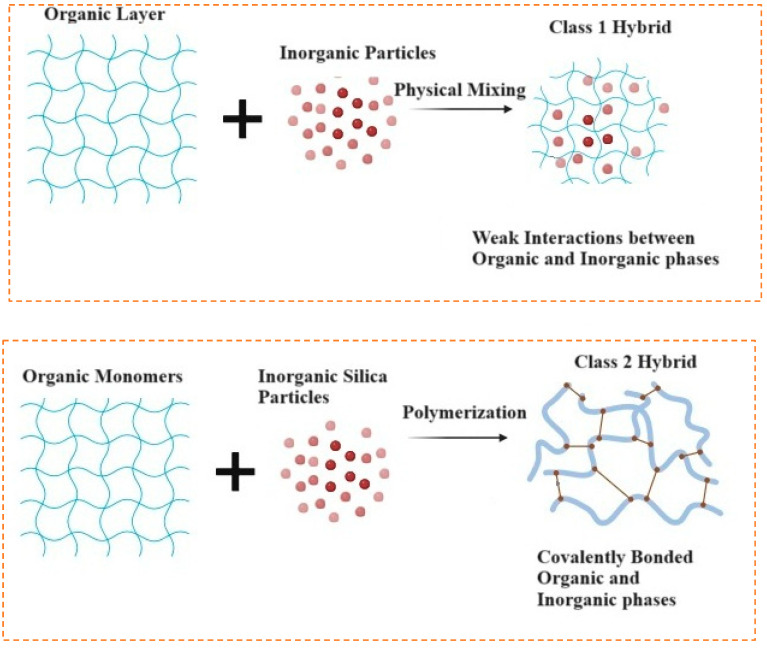
Class 1 and Class 2 hybrid materials. Created in Bio Render. Tahsin, K. (2025) https://BioRender.com/vs3l7cq (accessed on 1 November 2025).

(O/I) hybrid biomaterials are defined as organic and inorganic materials combined at a molecular level, with their phases being indistinguishable at the nanoscale and above [126]. These hybrids are formed by interpenetrating networks of organic and inorganic biomaterials interacting below the nanoscale. Unlike nanocomposites, where phases remain distinct, the phases in O/I hybrids blend seamlessly at the nanoscale [127]. These biomaterials show homogeneous dispersion of organic and inorganic components, either as building blocks or interwoven networks. Due to their highly organized molecular structure, hybrid biomaterials not only exhibit the intrinsic physical properties of both organic and inorganic components, but also display new properties arising from their synergistic effects [128]. To mix the organic and inorganic components at the molecular level, low-temperature synthesis methods, such as the sol–gel process, are typically used. The close molecular interactions between the phases enable the O/I hybrid material to function as a unified material with customizable mechanical, chemical, and physical properties [129]. However, due to the differing chemical nature of the organic and inorganic components, phase separation can occur during synthesis if there are no reactive sites in both phases. Therefore, it is necessary to select appropriate polymers or functionalize the polymer before synthesizing hybrid biomaterials that incorporate bioactive glass (BG) as the inorganic component. Based on the nature of interactions between the phases, hybrid materials are divided into two categories: Class I hybrids, which have weak molecular interactions like van der Waals forces, hydrogen bonding, or weak electrostatic interactions, and Class II hybrids, which have strong chemical interactions such as covalent bonds between the components [126], as shown in Figure 8. This review will focus on Class 2 Hybrid biomaterials and their use as biomaterials in bone tissue engineering.

### 5.4. Implant Coatings Made from Bioactive Glass

**Figure 9:**
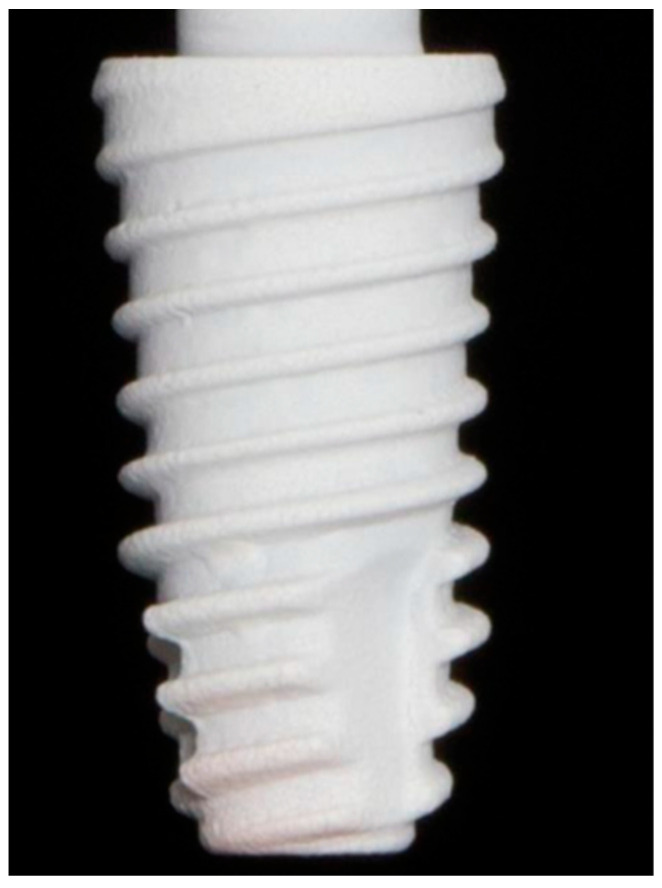
BG coating on a titanium implant screw via electrophoretic deposition [140]. (Adapted from https://creativecommons.org/licenses/by/4.0/ (accessed on 1 November 2025)).

Implant surfaces showed favorable biocompatibility and enhanced bone healing performance after coating with bioactive inorganic materials [130]. The coatings had been completed on titanium and zirconia implants [131]. HA was used in dental implants because of its bioactive characteristics that emulate genuine bone. Hyaluronic acid is being extensively used in dental surgery for bone implants due to its chemical characteristics [132]. After surgery, the material’s phosphate and calcium ion content resulted in low toxicity when implanted. Furthermore, a calcium-deficient layer that defined the contact between the bone and the implant promoted the direct bonding of the resulting bone structure to HA after surgery. While hydroxyapatite (HA) is a promising biomaterial for dental applications, due to its biocompatibility and ability to mimic natural tooth structure, some challenges exist, including potential for bacterial susceptibility, coating failure, and the need for further research on long-term effectiveness and optimal particle size [133]. Bioactive glass, as seen in Figure 9, had also been used to cover implants before HA. More bioactivity, osteoblast metabolic activity, bone regrowth, and antibacterial properties were seen in implants with bioactive glass layers [134,135,136,137]. In plasma-sprayed silicates of calcium coatings in [138], calcium was replaced with magnesium, zinc, and strontium ions. It was found that doping with these charged particles enhanced the biological qualities and decreased the degradative behavior. Silicate coating improved the bactericidal activity, binding strength, surface roughness, and degradation rate of metallic implant surfaces [139].

Coatings and materials for orthopedic or dental materials by various processes, such as hydrothermal [141], mechanochemical [142], precipitation [143], hydrolysis [144], and sol–gel methods [145].

#### 5.4.1. Coating Synthesis

Various methods can be employed to synthesize organic–inorganic hybrid materials [146]:

- Sol–gel process;
- In situ polymerization;
- Chemical vapor deposition (CVD);
- Hydrolysis.

#### 5.4.2. Sol–Gel Coating Process

The sol–gel approach, a versatile method for synthesizing inorganic materials, involves transforming liquid solutions (sol) into solid three-dimensional gel structures [147,148]. This technique enables precise control over the chemical composition and properties of the final material. In the case of organic–inorganic hybrids produced via the sol–gel method, key factors such as reaction parameters, precursors, and plant components play a crucial role. It is well-established that altering reaction conditions—such as time, temperature, and concentration—while using the same precursors can yield materials with distinct morphologies and properties. The structure of the biomaterial can be tailored and controlled to suit specific biomedical applications, including gels, powders, films, glasses, or ceramics.

For bone compatibility applications, SiO_2_–P_2_O_5_–CaO-based tertiary bioactive glasses, prepared through sol–gel processes, have been extensively used due to their biocompatibility, osteoconductivity, biodegradability, and ability to form bone-like mineral phases [149]. The ideal bioactive glass composition was kept constant as 70 mol % SiO_2_, 26 mol % CaCl_2_, and 4 mol % P_2_O_5_ for bone regeneration applications [150]. Despite their excellent in vitro and in vivo performance, their brittle and stiff nature imposed challenges for processing them into porous complex scaffolds, and their rapid degradation caused insufficient bone regeneration [151].

**Figure 10:**
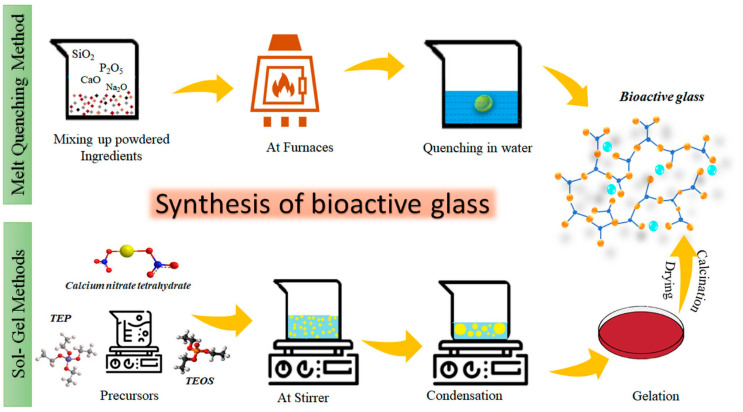
Different methods of synthesis of bioactive glass in the sol–gel process (adapted from [152], licensed under CC-4.0 https://creativecommons.org/licenses/by/4.0/).

Polydimethoxysilane (PDMS) is a polymer that contains functional silane groups within its backbone. It has been utilized to create class II hybrids through hydrolysis with tetraethylorthosilicate (TEOS). However, PDMS has limited use in tissue engineering, due to its non-degradable nature. An alternative approach to introducing silane functional groups into polymer chains is by copolymerizing a monomer with an alkoxysilane monomer. Copolymers such as polystyrene, poly(2-hydroxyethyl methacrylate), acrylonitrile butadiene styrene, and poly(methyl methacrylate) have been synthesized using various trialkoxysilyl (Si(OR)_3_) monomers. These copolymers, along with their corresponding hybrids formed through hydrolysis with silica precursors, are not biodegradable or leachable in bodily fluids, which limits their potential for bone regeneration applications. Previously published in our lab, we have prepared copolymers of vinylpyrrolidone (VP) and triethoxyvinylsilane (TEVS), which were then hydrolyzed and polycondensated with tetraethyl orthosilicate (TEOS) and triethyl phosphate (TEP) in an aqueous sol–gel process, using ethanol as the solvent to achieve a homogeneous organic/inorganic (O/I) network formation [150]. The prepared hybrid was proven to be degradable and cytocompatible, enabling bone regeneration. Despite their rapid degradation and excellent compatibility in bone regeneration, they lacked the ability to reduce infections on site, which could lead to potential implant failure. Figure 10 shows two different methods of sol gel processes and below is a description of the *sol–gel* process:

- **Preparation of the *sol***

A precursor (typically metal alkoxides or inorganic salts) is dissolved in a solvent. Hydrolysis and condensation reactions occur, forming a colloidal solution (sol). An inorganic matrix network forms and starts to gel.

- **Gelation**

The sol undergoes polymerization, creating a three-dimensional network. The system transitions into a gel-like structure with interconnected solid and liquid phases.

- **Aging and drying**

The gel is aged to strengthen its network. Drying removes solvents, resulting in a porous or dense solid, depending on the process.

- **Thermal treatment (if required)**

Additional heat treatment can be applied to remove organic residues or improve crystallinity. This step is common in the fabrication of ceramics and glasses.

#### 5.4.3. Advantages of the Sol–Gel Process [153,154,155]

**Figure 11:**
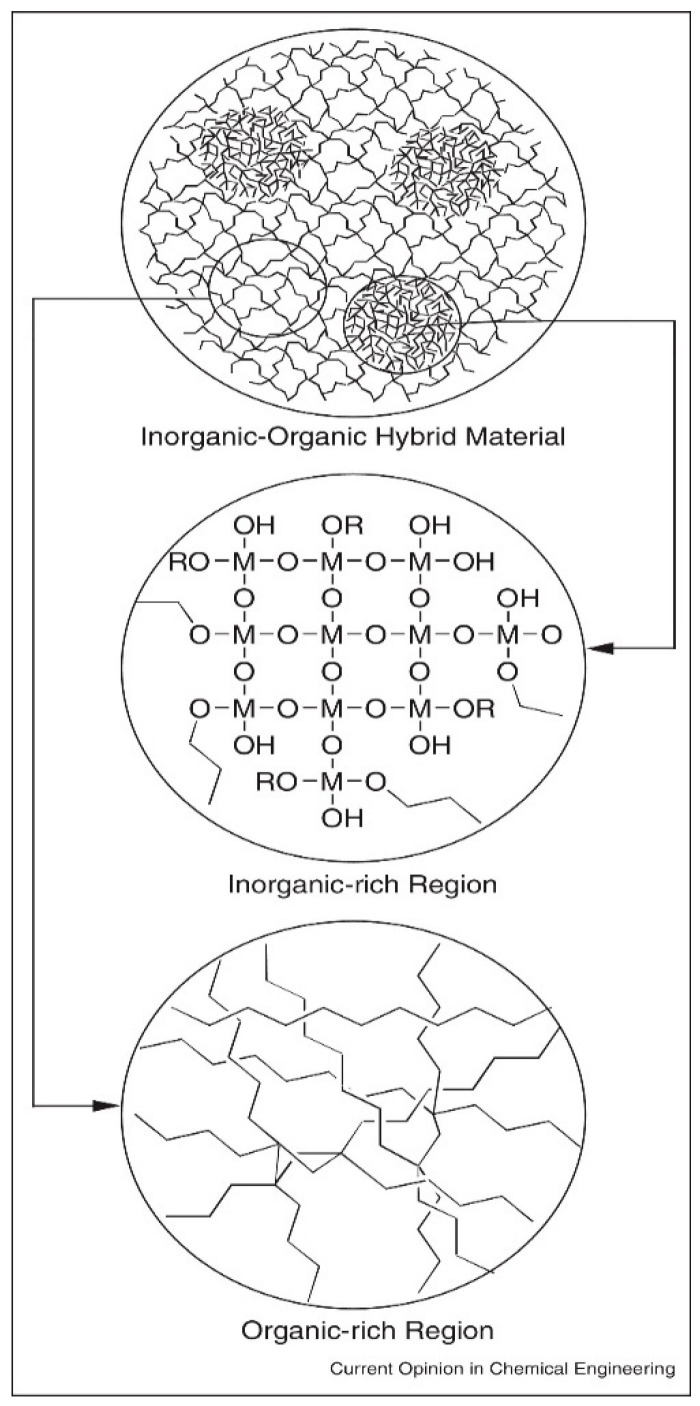
A combination of the two phases explains the term ‘ceramer’, which is often associated with inorganic–organic hybrid. (Adapted with permission from [156]).

- Precise control: Allows fine-tuning of material composition and properties.
- Simple/efficient: Suitable for applications where high temperatures may degrade components. Very high production efficiency. Low initial investment while having high quality products.
- Versatility: Can produce various material forms (thin films, coatings, fibers, and powders).
- Purity and homogeneity: Ensures uniform chemical distribution of organic and inorganic phases as shown in Figure 11.

#### 5.4.4. Combination of the Sol–Gel Method with Coating Techniques

**Dip coating:** The sol–gel method is a chemical approach used to synthesize solid materials from liquid solutions. In contrast, the dip-coating technique entails submerging an object in a liquid solution and then allowing it to dry, forming a thin film on its surface. Incorporating sol–gel solutions into the dip-coating process represents the application of sol–gel technology within this technique [157].

Depending on the desired properties of the final film, the rate of immersion and duration within the sol–gel solution influence the coating’s thickness. After submersion, the coated surface is removed from the solution, allowing the gel to adhere to its surface by forming hydrogen bonds or other bonds. The coating is then dried, either through air drying or by using a temperature-controlled oven. During this process, the gel solidifies into a stable layer.

**Figure 12:**
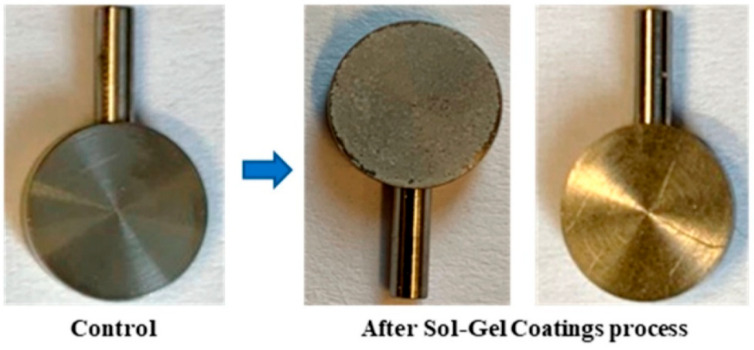
Sol–gel coating on titanium grade 4 substrate (reused with permission from [158] licensed under CC-BY-NC-ND 4.0: https://www.mdpi.com/openaccess (accessed on 1 November 2025)).

Thin coatings with a range of properties, including chemical and thermal resistance, transparency, and corrosion resistance, may be produced by combining the sol–gel process with the dip-coating method (Figure 12). This combination is applicable to a wide range of industries, such as the semiconductor sector, the manufacturing of specialty glass, corrosion prevention, and other sectors needing specific coating qualities, such as the biomedical sector, where materials are coated with sol–gel solutions to gain antibacterial, antioxidant, and anti-inflammatory qualities [157].

**Spin-coating:** This is another technique which entails rapidly spinning the sol–gel solution after it has been poured onto an implant surface. The centrifugal force of the rotation pushes the liquid outward, coating the substrate with a thin, uniform layer [159]. Spin-coating entails rapidly spinning a liquid solution containing the required component after it has been poured onto a flat surface, which is often a substrate. Spin-coating produces a very homogeneous layer thickness of a few nanometers to a few microns. The key advantage over other methods is its quick and simple deposit with an incredibly uniform deposition of various nanostructured materials’ films. Spin-coating makes it possible to apply a very homogeneous layer with a comparatively adjustable and repeatable thickness across a wide area on a flat substrate. Inorganic, organic, and inorganic/organic solution mixes can all be coated via spin-coating. With this method, spin-coating is quite resilient because the only factors that can be changed are spin speed and fluid viscosity. Both methods offer advantages and disadvantages when it comes to manufacturing layer uniformity, thickness control, and material compatibility. The use of spin-coating over any other method of Ti alloys via the sol–gel process was determined by the requirements of the application and the desired characteristics of the deposited layer.

**3D printing:** By integrating the advantages of 3D printing with the unique properties of sol–gel materials, this approach enables the fabrication of three-dimensional structures with tailored characteristics [160]. To enhance printability, the sol–gel solution can be modified with specialized additives, such as binding agents, rheological modifiers, or other compounds. The 3D printer is loaded with the sol–gel solution, which is then deposited layer by layer, following a predefined path to build the desired object. After printing, the object may require drying to remove moisture and initiate the curing process.

### 5.5. Sol–Gel-Based Antimicrobial Materials

Antibacterial materials are drawing more and more attention from researchers, particularly when they can be infused into bioactive materials. To construct future bioactive materials, designs have combined several organic molecules, such as polyethylene glycol, heparin, dextran sulfate, nafion, or polystyrene sulfonate, with TEOS as the main inorganic precursor to generate antimicrobial materials based on silica. The sol–gel process can be used to provide materials with antibacterial qualities to create surfaces that inhibit the development of bacteria and biofilm. Enhancing the antibacterial impact of bioactive glass has emerged as a prominent area of current study. Researchers in [161] created Ag containing bioactive glass, for dental restorations that were effective against *Streptococcus mutans.* Functionalizing a silica–Poly(vinylpyrrolidone) hybrid with small molecules like vancomycin and ciprofloxacin was found to be antibacterial against *S. aureus*, *Bacillus cereus*, *E. coli*, and *Pseudomonas aeruginosa* [162]. Other hybrid thin films based on polyvinyl alcohol (PVA)/tetraethyl orthosilicate (TEOS) and embedded with silver nanoparticles (AgNps) were synthesized using the sol–gel method and showed bactericidal effects against *E. coli*, *S. aureus*, and *P. aeruginosa* [163].

QASs in combination with the bioactive glass are another line of development. The antibacterial agent dodecyl-di(aminoethyl)-glycine (DDAG) was incorporated using the dip-coating method during the deposition of TEOS-derived xerogel films onto a glass substrate. The colony-forming units (CFU) of *E. coli*, *S. aureus*, and *P. aeruginosa* on the antibacterial-coated glass decreased by more than 99% when 1% of the antimicrobial agent was added to the coating solution, compared to glass coated without the antimicrobial agent [164]. Stainless steel was coated with a sol–gel film containing 40% N-(6-aminohexyl)-amino propyl trimethoxy silane and 60% butyl trimethoxy silane. NO-releasing coatings significantly reduced bacterial attachment, while untreated and sol–gel-coated steel showed similar adherence levels [165].

Incorporating antimicrobial compounds into sol–gel coatings has proven highly effective in preventing biocontamination and microbial growth [166]. Extensive research is needed to further explore the antibacterial, antifungal, and antiviral properties of sol–gel-based bioactive coatings. Antimicrobial sol–gel materials intended for dental applications require comprehensive evaluation to elucidate their linkage chemistry, which directly influences coating stability, durability, and the sustained antimicrobial effect at the implant interface. It is essential to distinguish genuine contact-killing mechanisms from potential leaching or release-based activity, as uncontrolled release may compromise both antimicrobial longevity and biocompatibility. Furthermore, systematic assessment following iocompatibility standards, along with essential in vivo endpoints—including peri-implant tissue response, osseointegration, systemic toxicity, and long-term antimicrobial persistence—is crucial to establish their safety, efficacy, and clinical applicability in the oral environment.

## 6. Gaps and Future Directions

Grade 4 titanium has long been the metal substrate standard for dental implants due to its favorable mechanical properties and well-documented clinical success. It has been extensively studied worldwide, with numerous reports confirming its reliability and safety in dental applications. Compared to lower-grade titanium alloys, Grade 4 titanium offers higher tensile strength and reduced malleability, making it suitable for standard dental implants. However, it is generally not recommended for narrow-diameter implants or orthopedic prostheses, where higher mechanical loads are encountered. Despite its widespread use, Grade 4 titanium is often critiqued for certain limitations, including relatively poor wear resistance, lower biocompatibility, and a higher Young’s modulus, which may contribute to stress shielding in the surrounding bone tissue. Titanium does not chemically bond with bone, leading to implant loosening or failure [61]. In order to enhance osseointegration by promoting bone growth around the implant, other bioactive treatments or layers must be added. For example, it has been shown that surface roughness influences the interactions between the implant and the bone [61]. Due to this, a number of studies have tried to create changed surfaces using physical and chemical methods (such as sandblasting, acid etching, blasting and etching combined, electrochemical oxidation, and laser treatments).

To further alter the implant surfaces and enhance the functionality of metallic implants, coating materials may also be used. While the mass of the implant greatly influences the mechanical qualities, the material surface characteristics are critical in the chemical and biological interactions with the surrounding bone tissue [61].

In 1969, Larry Hench and co-workers brought into the market, chemically bone-alike Hench’s 45S5 Bioglass^®^ based on 45% SiO_2_, 24.5% Na_2_O, 24.5% CaO, and 6% P_2_O_5_. tested in a rat femoral implant [167]. Si(OH)_4_ reacted to create a hydrated mesh of silica gel. A day later, Ca, PO_4_, and CO_3_ precipitated on the top layer of silica gel, creating the carbonate apatite foundation. A skeletal matrix eventually formed as a result of the accumulation of macrophages and differentiated stem cells in the carbonated apatite. At last, the matrix solidified, improving bone development [168].

The FDA has approved bioactive glass (Bioglass^®^ 45S5 and S53P4) for clinical applications. Bioactive glasses are being used more frequently in dental restorative materials, toothpaste, mineralizing and desensitizing agents, pulp capping, root canal therapy, and air abrasion, due to FDA permission [169]. BG is the best choice for dental applications due to its hemostatic effect on trabecular bone, dentin remineralization, and the removal of enzymatic degradation at the dentin interface, and they are used in periodontal surgical treatments to promote bone regeneration, particularly in inter-proximal bone defects [169]. It is reported that these two formulations of BG S53P4 and 45S5 were antimicrobial and had antibiofilm properties, against pathogens of osteomyelitis: *Staphylococcus aureus*, *Staphylococcus epidermidis*, *Enterococcus faecalis*, *Escherichia coli*, and *Candida albicans* [170]. This targets a limited range of species (only Gram-positive), which can miss emerging or less-studied pathogens. Incorporating antimicrobials into BG will address the need for a broader spectrum of antimicrobial BG. Translating antimicrobial dental materials from bench to clinic will increasingly require not only convincing efficacy but also a clear regulatory and commercialization strategy and a plan for integration into modern digital workflows. Regulatory bodies emphasize rigorous biological evaluation (ISO 10993 series) and material safety documentation as central requirements for any novel dental material, including antimicrobial coatings and impregnated polymers; sponsors should therefore design preclinical studies to address cytotoxicity, sensitization, systemic toxicity, and long-term exposure, consistent with FDA guidance and ISO 10993 best practice.

In regions subject to the European Medical Device Regulation (MDR) and related MDCG guidance, manufacturers must also anticipate stricter device classification, clinical evidence expectations, and technical documentation for materials that actively modify the local microbiology (for example, leachable antimicrobials or surfaces intended to kill microbes). Early engagement with notified bodies and clear clinical performance endpoints will shorten review timelines and reduce rework during conformity assessment.

Antimicrobial sol–gel materials designed for dental implant coatings require comprehensive evaluation to ensure both biological safety and mechanical reliability. Detailed characterization of linkage chemistry is critical, as it dictates coating stability, interfacial bonding, and the sustained antimicrobial function under oral conditions. It is essential to distinguish between true contact-killing mechanisms and potential leaching or release-based activity, as uncontrolled release can compromise long-term efficacy and biocompatibility.

In accordance with the ISO 10993 series, a biocompatibility assessment should include cytotoxicity (ISO 10993-5 [171]), sensitization (ISO 10993-10 [172]), genotoxicity (ISO 10993-3 [173]), and implantation studies (ISO 10993-6 [174]), along with systemic toxicity evaluations (ISO 10993-11 [175]). For coatings, adhesion strength and fatigue behavior are critical determinants of long-term clinical performance; these may be assessed following ISO 2409 [176] (cross-cut adhesion test), ASTM D4541 [177] (pull-off adhesion test), and ISO 14801 [178] (fatigue testing for endosseous dental implants).

Antibacterial efficacy should be evaluated following standardized methods such as ISO 22196 [179] or JIS Z 2801 [180] for surface antibacterial activity, and where relevant, dynamic biofilm assays under simulated oral conditions. Additional mechanical assessments, including wear, corrosion resistance, and coating delamination under cyclic loading, provide further insight into the material’s reliability in vivo.

Ultimately, integrating these biological and mechanical evaluations—alongside essential in vivo endpoints, such as peri-implant tissue response, osseointegration, and long-term antimicrobial persistence—is crucial to validate the safety, efficacy, and translational potential of antimicrobial sol–gel coatings for dental implant applications.

The addition of Zn^+2^ and Cu^+2^ ions in sol–gel-based bioactive glasses remains the most common approach to combine antimicrobial effects to BG. As mentioned in Section 4.2 (Table 1 and Table 3), the release of these metal ions from the bioactive glasses and its related cytocompatibility with osteoblast-like cells has been reported [56]. Ag-doping, even at low concentrations, was not cytotoxic for osteoblast in vitro [55] but studies showed that Ag^+^, Zn^2+^, and Hg^2+^ ions are very cytotoxic, even at low concentrations [56]. While Cu ions may promote osteoblast proliferation, differentiation, and migration [63], high concentrations of Cu ions inhibit growth and cause cell death and toxicity in humans [64]. Although significant progress has been made in the development of antimicrobial additions to BG, further comprehensive studies are required to better understand the incorporation and release behavior of antimicrobial agents, as well as their overall biological performance.

In particular, the impact of such modifications on antimicrobial efficacy and cellular responses remains an area of active investigation. Therefore, the primary objective of this study is to synthesize ternary and quaternary sol–gel-derived bioactive glass systems, incorporating a variety of antimicrobial agents. These hybrid materials are designed to enhance antimicrobial activity while maintaining compatibility with biological tissues. In this context, the antimicrobial properties of the synthesized glasses were assessed, and their cytocompatibility was evaluated using osteoblast-like cells to determine their potential for applications in bone tissue engineering and infection prevention.

QAS are antimicrobials with the broadest spectrum of activities, as reported in Section 4.4. QACs are lethal to a wide variety of organisms, Gram-positive and Gram-negative bacteria, fungi, parasites (e.g., *Leishmania major*, *Plasmodia falciparum*), and lipophilic (enveloped) viruses [181]. These stable, non-leaching antibacterial materials offer prolonged antimicrobial efficacy through direct contact between microorganisms and the biocidal surface without compromising the mechanical integrity or polymerization characteristics of the original non-antibacterial dental formulations. Notably, quaternary ammonium (QA)-based resin materials demonstrate favorable biocompatibility, as indicated by their low toxicity, minimal allergenic potential, and limited tissue irritability [181]. Given these advantageous properties, quaternary ammonium compounds (QACs) are considered to be highly promising for the prevention and management of dental caries. QASs could be used in combination with the bioactive glass and are currently another line of development. The only QAS was dodecyl-di(aminoethyl)-glycine (DDAG), which was incorporated using the dip-coating method during the deposition of TEOS-derived xerogel films onto a glass substrate. The colony-forming units (CFU) of *E. coli*, *S. aureus*, and *P. aeruginosa* on the antibacterial-coated glass decreased by more than 99% when 1% of the antimicrobial agent was added to the coating solution, compared to glass coated without the antimicrobial [164]. Stainless steel was coated with a sol–gel film containing 40% N-(6-aminohexyl)-amino propyl trimethoxy silane and 60% butyl trimethoxy silane. NO-releasing coatings significantly reduced bacterial attachment, while untreated and sol–gel-coated steel showed similar adherence levels [165]. The development of advanced biomaterials design—particularly the rise in smart materials—encourages the need for novel opportunities in the formulation of bioactive glass materials with quaternary ammonium (QA)-functionalized. This approach may address limitations and challenges associated with integrating antibacterial agents in their conventional (bulk) forms into such materials. Incorporating antimicrobial compounds into sol–gel coatings has proven highly effective in preventing biocontamination and microbial growth [166]. Extensive research is needed to further explore the antibacterial, antifungal, and antiviral properties of sol–gel-based bioactive coatings.

Generally speaking, QASs’ additional carbon chain permits more structural modifications than other antimicrobial agents, and additional functional groups can be added based on the needs of the application. For instance, an increase in biocompatibility or alterable toxicity is required in terms of medical applications.

Despite encouraging in vivo studies demonstrating the feasibility of using quaternary ammonium salts (QASs) in dental applications, their potential adverse effects remain a significant barrier to dental/clinical translation. Therefore, strategies to mitigate or eliminate these undesirable effects warrant careful consideration. We propose three potential approaches to address this challenge.

First, QASs can be combined with low-toxicity materials—bioactive glass is a notable example. QAS-incorporated hydrogels have recently exhibited excellent therapeutic efficacy and high biocompatibility in in vivo studies [92].

To further enhance their safety profile, linkages to degradable materials can enhance the biodegradation of newly synthesized QAS [182].

Addition to metal substrates will help enhance tensile strength, load-bearing capacity, and quality.

Furthermore, using QAS hybrids and coatings with low-toxicity materials, macromolecules can be cross-linked to form finer shapes, such as microspheres and three-dimensional mesh structures, to improve the comprehensive properties of the material.

Commercialization challenges remain substantial. Key hurdles include the scale-up of reproducible coatings/resins, ensuring manufacturing processes do not alter antimicrobial or mechanical properties, demonstrating durable performance under oral wear/fatigue, and addressing safety/toxicity concerns (including potential systemic exposure or contribution to resistance). Economic challenges—raw material cost, additional quality-control testing, and longer time-to-market for combination (device + antimicrobial) products—increase capital requirements and can deter SMEs. Past studies on translational bottlenecks in advanced biomaterials highlight funding timelines, regulatory uncertainty, and the need for cross-disciplinary teams (engineering, regulatory, clinical, commercial) to succeed.

Digital dentistry (CAD/CAM and additive manufacturing) offers an important route for rapid clinical translation and individualized antimicrobial solutions. Printable antimicrobial resins and surface functionalization compatible with stereolithography or selective laser processes enable patient-specific restorations with integrated bioactive function (e.g., antimicrobial nanoparticles or covalently bound antimicrobial peptides). However, integrating antimicrobial chemistries with printable materials adds complexity: photopolymerization kinetics, post-processing, and long-term leachability must be validated for both antimicrobial efficacy and mechanical performance. As additive manufacturing becomes routine in clinics, regulatory expectations for printed, antimicrobial-functionalized devices will require a demonstration of process control, batch reproducibility, and device-specific biocompatibility.

Scientific and commercial opportunities for the coming 5–10 years include (1) contact-killing surface chemistries that avoid sustained release and therefore reduce systemic exposure and selective pressure for resistance; (2) smart, stimuli-responsive systems that release antimicrobials only when biofilm formation is detected; (3) combination approaches that pair antimicrobial surface properties with biofilm-disrupting enzymes or quorum-sensing inhibitors; and (4) modular antimicrobial “additives”, formulated for seamless integration into existing CAD/CAM and 3D-printing workflows. However, each innovation must be accompanied by standardized, clinically relevant testing (long-term wear, salivary enzymes, repeated contamination cycles) and by engagement with regulators and clinical end users early in development.

In summary, a successful path to clinic for antimicrobial dental materials requires the following: concurrent planning for regulatory evidence (ISO 10993 endpoints and device-specific clinical data), realistic commercialization planning (scale-up, costs, supply chain, IP strategy), and a technical design that accounts for digital dentistry workflows. Explicitly addressing these areas in preclinical programs and early clinical studies will increase the probability of adoption and safe, durable patient benefit.

## 7. Conclusions

This review included relevant background information on biocides and antimicrobial materials that are compatible in promoting healing, along with effective antimicrobial properties. Despite the fact that antibacterial materials are also utilized in implants, the subject of antimicrobial materials is so vast that it was not feasible to cover every facet in this brief overview. Nonetheless, every attempt was made to minimize bias and incorporate noteworthy research findings and vital work. This paper discussed advancements, commonly associated materials, cytotoxic studies, antimicrobial efficacy, and constraints on the development of antimicrobial material for coating implant surfaces, in addition to the numerous opportunities and problems presented by bone tissue engineering techniques. This review examines the antimicrobial mechanisms of various agents while highlighting the influence of applications, the synthesis process, and limitations.

## Figures and Tables

**Table 1:** Summary of studies investigating the microbiology of failing implants (Reused with permission from [33]. Copyright © 2009, Elsevier).

Type of Implant (No. of Patients/Implants)	Most Prevalent Microbes Detected (% Sites Infected with Bacteria)
**Brånemark:** System is a well-established and widely used dental implant system based on the principle of osseointegration. The original Brånemark implant was a cylindrical, pure titanium implant with smooth, polished screw-like threads.	*Prevotella intermedia/P. nigrescens* 60% *Actinobacillus actinomycetemcomitans* 60% *Staphylococci*, *coliforms*, *Candida* spp. 55%
Not stated.	*Bacteroides forsythus* 59% *Spirochetes* 54% *Fusobacterium* spp. 41% *Peptostreptococcus micros* 39% *Porphyromonas gingivalis* 27%
Titanium hollow cylinder implants (7/not stated).	*Bacteroides spp.*, *Fusobacterium* spp., spirochetes, fusiform bacilli, motile and curved rods (% not stated)
Not stated (13/20).	*Staphylococcus* spp. 55%
Not stated (21/28).	*P. nicrescens*, *P. micros*, *Fusobacterium nucleatum* (% not stated)
**IMZ:** The IMZ (IntraMobil Zylinder) implant system was notable for its two-part design, which included an inner elastic intramobile element that aimed to mimic the natural flexibility of teeth. This design was meant to reduce stress on the bone and improve load distribution. However, IMZ implants are now considered outdated and are rarely used in modern implants.	*Bacteroides* spp. 89% *Actinobacillus actinomycetemcomitans* 89% *Fusobacterium nucleatum* 22% *Capnocytophaga* spp. 27.8% *Eikenella corrodens* 17%
**Astra:** Widely used in implant dentistry by OsseoSpeed™ surfaces, in micro thread technology with conical design, reducing complications like peri-implantitis. Astra implants come in various lengths and diameters, making them versatile for different clinical cases, including single tooth replacement, multiple teeth, and full-arch reconstructions. **ITI Staumann**: Made of a titanium–zirconium alloy that is stronger than pure titanium, allowing for smaller implants with high strength—ideal for patients with limited bone. SLActive^®^ Surface, modified hydrophilic implant surface speeds up osseointegration, reducing healing time. Esthetic finishing in visible areas. MorsTaper Connection for antimicrobial effects.	*Actinomyces* spp. 83% *F. nucleatum* 70% *P. intermedia/nigrescens* group 60% *Steptococcus anginosus* (*milleri*) group 70% *P. micros* 63% *Enterococcus* spp. 30% *Yeast* spp. 30%

**Table 2:** Type of antimicrobial peptides used in dentistry [51].

PEPTIDE NAME	APPLICATION	DESCRIPTION
**LACTOPEROXIDASE**	Toothpaste, mouthwash, and gel	Used as a saliva substitute and showed improvement of xerostomic symptoms and a reduction in streptococci.
**GERM CLEAN**	Oral spray	Oral spray containing GERM CLEAN showed an inhibitory effect on the initial adhesion, acid production, extracellular polysaccharides production, and biofilm formation of *Streptococcus mutans*.
**C16G2**	Oral rinse	C16G2 oral rinse showed a decrease in plaque, salivary *S. mutans*, lactic acid production, and enamel demineralization.
**TET213**	Dental implant coating	CaP-Tet213 and CaP-HHC36 coating showed antimicrobial activity against *Staphylococcus aureus* and *Pseudomonas aeruginosa*.
**HHC36**		
**β-DEFENSIN-2**		Coated recombinant human β-defensin-2 on titanium surfaces yielded antimicrobial activities and prevented bacterial colonization.
**HUMAN Β-DEFENSIN-3 CONTAINING CHIMERIC PEPTIDES**		Chimeric peptide containing human β-defensing-3 coating prevented biofilm formation by inhibition of initial colonizing Streptococci.
**LL-37**		Nanopore coating loaded with LL-37 showed diverse antibacterial and osteogenic induction abilities.

**Table 3:** Type of metals, their properties, toxicity, and antimicrobial effectiveness.

Metal	Features	Toxicity Profile	Antimicrobial Ability
**Silver**	TiN/Ag-modified titanium alloy produced via multiarc ion-plating and ion implantation exhibited stable antimicrobial activity against *Staphylococcus epidermidis* for over 12 weeks in vitro [54]. A study using PIII to embed Ag into Ti, Si, and SiO_2_ surfaces found that electron transfer between Ag nanoparticles and Ti is the initial step in the antibacterial mechanism [55].	Silver at low concentrations was not cytotoxic for osteoblast in vitro [55]. Studies showed that Ag^+^, Zn^2+^, and Hg^2+^ ions are very cytotoxic, even at low concentrations [56].	Effective against *S. choleraesuis*, *E. coli* [57], *S. aureus*, and *S. epidermis* [58].
**Copper**	N/Cu-incorporated Ti produced by PIII showed strong antibacterial activity against *Staphylococcus aureus* and *Escherichia coli*, along with enhanced angiogenic properties from Cu and excellent corrosion resistance from TiN [59]. Another study found that the form of Cu (metallic Cu or CuNPs) in coatings depends on synthesis parameters, with metallic Cu showing superior antibacterial activity and biocompatibility compared to CuNPs [60]. The study emphasized that preparation technology parameters critically influence a surface’s antibacterial performance and biocompatibility.	Essential metal ion functioning of organs and metabolic processes [61]. Cu deficiency results in anemia, heart disease, arthritis, and osteoporosis, etc. [62]. Cu ion promotes osteoblast proliferation, differentiation, and migration [63]. High concentrations of Cu ions inhibit growth and cause cell death and toxicity on humans [64].	Effective against MRSA [65] and *E. coli* [66] within a few hours. Copper inhibited *K. aerogenes* [67] and *S. aureus* [65].
**Zinc**	Zinc, ZnO, nano ZnO, and Zn^2+^ ion release are antibacterial agents. Used as dental and formulated into dental hygiene products to control plaque, such as mouth rinses and toothpaste [68]. Ti surface with Zn–Ag increased ratio of Zn and made up for the inhibition of Ag on cell adhesion and growth of fibroblast-like cells [56].	Zn ion is not harmful to cells, and it has been known for a long time that zinc can help bones grow. Zinc is an important part of making DNA, enzymes working, nucleic acid processing, biomineralization, and hormone action [69].	Effective against *S. aureus*; *E. coli*; *S. choleraesuis* [55], *P. phosphoreum* [70], *and S. epidermis* [71].

**Table 4:** Common phytochemicals used in various medical applications against the targeted species.

Phytochemical	Material	Application	Antimicrobial Efficacy
***Malus domestica* L.**	Titanium implant coating [72].	Dental implantology	*Streptococcus mutans*, *Salmonella typhi* bacteria responsible for dental caries and periodontal diseases [73]. *Escherichia coli*, *Salmonella*, and *Listeria monocytogenes*
***Cissus quadrangularis* L.**	Periodontal filler in association with hydroxyapatite [74].	Periodontal regeneration	Gram-positive bacteria [75]: Bacillus subtilis, Bacillus cereus, Staphylococcus aureus, and Streptococcus species
***Carthamus tinctorius* L.**	Periodontal materials combined with a collagen sponge; periodontal filler integrated with a polylactide-glycolic acid bioresorbable barrier [76].	Periodontal regeneration.	*Escherichia coli* (*E. coli*), *Klebsiella pneumoniae* (*K. pneumonia*), *Acinetobacter baumannii* (*A. baumannii*), *Pseudomonas aeruginosa* (*P. aeruginosa*), *Staphylococcus aureus* (*S. aureus*) and *Salmonella* spp. [77]
***Glycine max* L.**	Bone filler [78].	Alveolar bone regeneration	*K. pneumoniae*, *L. monocytogenes* *S. aureus* [79]
**Chitosan**	The cell walls of fungal mycelia are composed of chitin, glucan, and glycoproteins. In species such as *Aspergillus niger*, *Mucor rouxii*, and *Penicillium notatum*, chitin can constitute up to 45% of the cell wall. Chitosan is produced by deacetylating chitin.	Guided tissue regeneration (GTR), hydrogel made of chitosan was developed with the purpose of delivering amelogenin, dentin bonding, and adhesion, coating of dental implants [80].	Prevents biofilm formation of *S. aureus*, *P. Aeruginosa*, *Proteus mirabilis*, and *E. coli* [81]. Antifungal against *Candida albicans*, *Candida tropicalis*, and other *Candida* species [82].
**Cannabidiol (CBD), derived from the Cannabis plant**	PMMA restorations.	To minimize denture-associated infections, antimicrobial enhancements to PMMA, the primary material for dentures, were coated with CBD nanoparticles [83].	Antimicrobial activity against the following: *Staphylococcus aureus*, *Escherichia coli*, *Streptococcus agalactiae* [83].

## Data Availability

No new data were created or analyzed in this study. Data sharing is not applicable to this article.

## Supplementary Materials

The following supporting information can be downloaded at: https://www.mdpi.com/article/10.3390/ma19020403/s1, S1. Review Methodology.

## References

[1] American Association of Oral and Maxillofacial Surgeons Oral and Maxillofacial Surgeons: The Experts in Face, Mouth and Jaw Surgery. https://myoms.org/what-we-do/.

[2] Gupta A., Dhanraj M., Sivagami G. (2010). Status of surface treatment in endosseous implant: A literary overview. Indian J. Dent. Res..

[3] Gaviria L., Salcido J.P., Guda T., Ong J.L. (2014). Current trends in dental implants. J. Korean Assoc. Oral Maxillofac. Surg..

[4] Lambris J.D., Paoletti R. Advances in Experimental Medicine and Biology. http://www.springer.com/series/5584.

[5] Magill S.S., Edwards J.R., Bamberg W., Beldavs Z.G., Dumyati G., Kainer M.A., Lynfield R., Maloney M., McAllister-Hollod L., Nadle J. (2014). Multistate Point-Prevalence Survey of Health Care–Associated Infections. N. Engl. J. Med..

[6] Montanaro L., Speziale P., Campoccia D., Ravaioli S., Cangini I., Pietrocola G., Giannini S., Arciola C.R. (2011). Scenery of *Staphylococcus* Implant Infections in Orthopedics. Future Microbiol..

[7] Li B., Webster T.J. (2018). Bacteria antibiotic resistance: New challenges and opportunities for implant-associated orthopedic infections. J. Orthop. Res..

[8] Gao J., Pan Y., Gao Y., Pang H., Sun H., Cheng L., Liu J. (2024). Research Progress on the Preparation Process and Material Structure of 3D-Printed Dental Implants and Their Clinical Applications. Coatings.

[9] Public Health Agency of Canada (2023). Pan-Canadian Action Plan on Antimicrobial Resistance.

[10] Zoutman D., McDonald S., Vethanayagan D. (1998). Total and Attributable Costs of Surgical-Wound Infections at a Canadian Tertiary-Care Center. Infect. Control Hosp. Epidemiol..

[11] Camps-Font O., Martín-Fatás P., Clé-Ovejero A., Figueiredo R., Gay-Escoda C., Valmaseda-Castellón E. (2018). Postoperative infections after dental implant placement: Variables associated with increased risk of failure. J. Periodontol..

[12] Gristina A.G. (1987). Biomaterial-Centered Infection: Microbial Adhesion Versus Tissue Integration. Science.

[13] Schierholz J.M., Beuth J. (2001). Implant infections: A haven for opportunistic bacteria. J. Hosp. Infect..

[14] Ivanovski S., Bartold P.M., Huang Y. (2022). The role of foreign body response in peri-implantitis: What is the evidence?. Periodontology 2000.

[15] Trindade R., Albrektsson T., Tengvall P., Wennerberg A. (2016). Foreign Body Reaction to Biomaterials: On Mechanisms for Buildup and Breakdown of Osseointegration. Clin. Implant Dent. Relat. Res..

[16] Thomas P., Summer B. (2017). Implant allergy. Allergol. Sel..

[17] Wiltshire W.A., Ferreira M.R., Ligthelm A.J. (2007). Allergies to dental materials. Vital.

[18] Schwarz F., Derks J., Monje A., Wang H.L. (2018). Peri-implantitis. J. Periodontol..

[19] Prathapachandran J., Suresh N. (2012). Management of peri-implantitis. Dent. Res. J..

[20] Hasan J., Bright R., Hayles A., Palms D., Zilm P., Barker D., Vasilev K. (2022). Preventing Peri-implantitis: The Quest for a Next Generation of Titanium Dental Implants. Am. Chem. Soc..

[21] Park Y.-S., Lee B.-A., Choi S.-H., Kim Y.-T. (2022). Evaluation of failed implants and reimplantation at sites of previous dental implant failure: Survival rates and risk factors. J. Periodontal Implant Sci..

[22] Zhang Y., Pajares A., Lawn B.R. (2004). Fatigue and damage tolerance of Y-TZP ceramics in layered biomechanical systems. J. Biomed. Mater. Res. B Appl. Biomater..

[23] Kashi A., Saha S. (2020). Failure mechanisms of medical implants and their effects on outcomes. Biointegration of Medical Implant Materials.

[24] Lambert F.E., Weber H., Susarla S.M., Belser U.C., Gallucci G.O. (2009). Descriptive Analysis of Implant and Prosthodontic Survival Rates With Fixed Implant–Supported Rehabilitations in the Edentulous Maxilla. J. Periodontol..

[25] Shetty P., Yadav P., Tahir M., Saini V. (2016). Implant Design and Stress Distribution. Int. J. Oral Implantol. Clin. Res..

[26] Liaw K., Delfini R.H., Abrahams J.J. (2015). Dental Implant Complications. Semin. Ultrasound CT MRI.

[27] Renvert S., Persson G.R., Pirih F.Q., Camargo P.M. (2018). Peri-implant health, peri-implant mucositis, and peri-implantitis: Case definitions and diagnostic considerations. J. Clin. Periodontol..

[28] Atieh M.A., Alsabeeha N.H.M., Faggion C.M., Duncan W.J. (2013). The Frequency of Peri-Implant Diseases: A Systematic Review and Meta-Analysis. J. Periodontol..

[29] Radaic A., Kapila Y.L. (2021). The oralome and its dysbiosis: New insights into oral microbiome-host interactions. Comput. Struct. Biotechnol. J..

[30] Lu M., Xuan S., Wang Z. (2019). Oral microbiota: A new view of body health. Food Sci. Hum. Wellness.

[31] Bolukbasi N., Ozdemir T., Oksuz L., Gurler N. (2012). Bacteremia following dental implant surgery: Preliminary results. Med. Oral Patol. Oral Cir. Bucal.

[32] Pye A.D., Lockhart D.E.A., Dawson M.P., Murray C.A., Smith A.J. (2009). A review of dental implants and infection. J. Hosp. Infect..

[33] Cheng G., Zhang Z., Chen S., Bryers J.D., Jiang S. (2007). Inhibition of bacterial adhesion and biofilm formation on zwitterionic surfaces. Biomaterials.

[34] Pang C.M., Hong P., Guo H., Liu W.-T. (2005). Biofilm Formation Characteristics of Bacterial Isolates Retrieved from a Reverse Osmosis Membrane. Environ. Sci. Technol..

[35] Goldberg J. (2002). Biofilms and antibiotic resistance: A genetic linkage. Trends Microbiol..

[36] Peng J.-S., Tsai W.-C., Chou C.-C. (2002). Inactivation and removal of *Bacillus cereus* by sanitizer and detergent. Int. J. Food Microbiol..

[37] Chen M.J., Zhang Z., Bott T.R. (1998). Direct measurement of the adhesive strength of biofilms in pipes by micromanipulation. Biotechnol. Tech..

[38] Dickinson G.M., Bisno A.L. (1989). Infections associated with indwelling devices: Infections related to extravascular devices. Antimicrob. Agents Chemother..

[39] Muller E., Hübner J., Gutierrez N., Takeda S., Goldmann D.A., Pier G.B. (1993). Isolation and characterization of transposon mutants of *Staphylococcus epidermidis* deficient in capsular polysaccharide/adhesin and slime. Infect. Immun..

[40] Mack D., Fischer W., Krokotsch A., Leopold K., Hartmann R., Egge H., Laufs R. (1996). The intercellular adhesin involved in biofilm accumulation of *Staphylococcus epidermidis* is a linear beta-1,6-linked glucosaminoglycan: Purification and structural analysis. J. Bacteriol..

[41] Sutherland I. (2001). The biofilm matrix—An immobilized but dynamic microbial environment. Trends Microbiol..

[42] Dunne W.M. (2002). Bacterial Adhesion: Seen Any Good Biofilms Lately?. Clin. Microbiol. Rev..

[43] Aboelnaga N., Elsayed S.W., Abdelsalam N.A., Salem S., Saif N.A., Elsayed M., Ayman S., Nasr M., Elhadidy M. (2024). Deciphering the dynamics of methicillin-resistant *Staphylococcus aureus* biofilm formation: From molecular signaling to nanotherapeutic advances. Cell Commun. Signal..

[44] Korber D.R., Lawrence J.R., Lappin-Scott H.M., Costerton J.W. (1995). Growth of Microorganisms on Surfaces. Microbial Biofilms.

[45] Silva R.C.S., Agrelli A., Andrade A.N., Mendes-Marques C.L., Arruda I.R.S., Santos L.R.L., Vasconcelos N.F., Machado G. (2022). Titanium Dental Implants: An Overview of Applied Nanobiotechnology to Improve Biocompatibility and Prevent Infections. Materials.

[46] Chen Z., Wang Z., Qiu W., Fang F. (2021). Overview of Antibacterial Strategies of Dental Implant Materials for the Prevention of Peri-Implantitis. Bioconjug. Chem..

[47] Li G., Jiang H., Yang F. (2022). A Novel Diffuse Plasma Jet Without Airflow and Its Application in the Real-Time Surface Modification of Titanium. IEEE Trans. Plasma Sci..

[48] Zhu J., Chu W., Luo J., Yang J., He L., Li J. (2022). Dental Materials for Oral Microbiota Dysbiosis: An Update. Front. Cell. Infect. Microbiol..

[49] An S.-J., Namkung J.-U., Ha K.-W., Jun H.-K., Kim H.Y., Choi B.-K. (2022). Inhibitory effect of d-arabinose on oral bacteria biofilm formation on titanium discs. Anaerobe.

[50] Masurier N., Tissot J.-B., Boukhriss D., Jebors S., Pinese C., Verdié P., Amblard M., Mehdi A., Martinez J., Humblot V. (2018). Site-specific grafting on titanium surfaces with hybrid temporin antibacterial peptides. J. Mater. Chem. B.

[51] Jain A., Dixit J., Prakash D. (2008). Modulatory effects of Cissus quadrangularis on periodontal regeneration by bovine-derived hydroxyapatite in intrabony defects: Exploratory clinical trial. J. Int. Acad. Periodontol..

[52] Wan R., Chu S., Wang X., Lei L., Tang H., Hu G., Dong L., Li D., Gu H. (2020). Study on the osteogenesis of rat mesenchymal stem cells and the long-term antibacterial activity of *Staphylococcus epidermidis* on the surface of silver-rich TiN/Ag modified titanium alloy. J. Biomed. Mater. Res. B Appl. Biomater..

[53] Wang G., Jin W., Qasim A.M., Gao A., Peng X., Li W., Feng H., Chu P.K. (2017). Antibacterial effects of titanium embedded with silver nanoparticles based on electron-transfer-induced reactive oxygen species. Biomaterials.

[54] Hardes J., Streitburger A., Ahrens H., Nusselt T., Gebert C., Winkelmann W., Battmann A., Gosheger G. (2007). The Influence of Elementary Silver Versus Titanium on Osteoblasts Behaviour In Vitro Using Human Osteosarcoma Cell Lines. Sarcoma.

[55] Heidenau F., Mittelmeier W., Detsch R., Haenle M., Stenzel F., Ziegler G., Gollwitzer H. (2005). A novel antibacterial titania coating: Metal ion toxicity and in vitro surface colonization. J. Mater. Sci. Mater. Med..

[56] Du W.-L., Niu S.-S., Xu Y.-L., Xu Z.-R., Fan C.-L. (2009). Antibacterial activity of chitosan tripolyphosphate nanoparticles loaded with various metal ions. Carbohydr. Polym..

[57] Xia C., Cai D., Tan J., Li K., Qiao Y., Liu X. (2018). Synergistic Effects of N/Cu Dual Ions Implantation on Stimulating Antibacterial Ability and Angiogenic Activity of Titanium. ACS Biomater. Sci. Eng..

[58] Yu L., Jin G., Ouyang L., Wang D., Qiao Y., Liu X. (2016). Antibacterial activity, osteogenic and angiogenic behaviors of copper-bearing titanium synthesized by PIII&D. J. Mater. Chem. B.

[59] Zhang J., Li Y., Yang K., Hao X. (2010). Effects of Cu^2+^ and Cu^+^ on the proliferation, differentiation and calcification of primary mouse osteoblasts in vitro. Chin. J. Inorg. Chem..

[60] Zhang D., Ren L., Zhang Y., Xue N., Yang K., Zhong M. (2013). Antibacterial activity against *Porphyromonas gingivalis* and biological characteristics of antibacterial stainless steel. Colloids Surf. B Biointerfaces.

[61] Palmquist A., Omar O.M., Esposito M., Lausmaa J., Thomsen P. (2010). Titanium oral implants: Surface characteristics, interface biology and clinical outcome. J. R. Soc. Interface.

[62] Staiger M.P., Pietak A.M., Huadmai J., Dias G. (2006). Magnesium and its alloys as orthopedic biomaterials: A review. Biomaterials.

[63] Noyce J.O., Michels H., Keevil C.W. (2006). Potential use of copper surfaces to reduce survival of epidemic meticillin-resistant *Staphylococcus aureus* in the healthcare environment. J. Hosp. Infect..

[64] Noyce J.O., Michels H., Keevil C.W. (2006). Use of Copper Cast Alloys To Control *Escherichia coli* O157 Cross-Contamination during Food Processing. Appl. Environ. Microbiol..

[65] Zevenhuizen L.P.T.M., Dolfing J., Eshuis E.J., Scholten-Koerselman I.J. (1979). Inhibitory effects of copper on bacteria related to the free ion concentration. Microb. Ecol..

[66] Almoudi M.M., Hussein A.S., Hassan M.I.A., Zain N.M. (2018). A systematic review on antibacterial activity of zinc against *Streptococcus mutans*. Saudi Dent. J..

[67] Li L., Li Q., Zhao M., Dong L., Wu J., Li D. (2019). Effects of Zn and Ag Ratio on Cell Adhesion and Antibacterial Properties of Zn/Ag Coimplanted TiN. ACS Biomater. Sci. Eng..

[68] Yamaguchi M., Oishi H., Suketa Y. (1987). Stimulatory effect of zinc on bone formation in tissue culture. Biochem. Pharmacol..

[69] Wang D., Lin Z., Wang T., Yao Z., Qin M., Zheng S., Lu W. (2016). Where does the toxicity of metal oxide nanoparticles come from: The nanoparticles, the ions, or a combination of both?. J. Hazard. Mater..

[70] Kokkonen H., Cassinelli C., Verhoef R., Morra M., Schols H.A., Tuukkanen J. (2008). Differentiation of Osteoblasts on Pectin-Coated Titanium. Biomacromolecules.

[71] Irshad A., Jawad R., Mushtaq Q., Spalletta A., Martin P., Ishtiaq U. (2025). Determination of antibacterial and antioxidant potential of organic crude extracts from *Malus domestica*, *Cinnamomum verum* and *Trachyspermum ammi*. Sci. Rep..

[72] Murthy K.N.C., Vanitha A., Swamy M.M., Ravishankar G.A. (2003). Antioxidant and Antimicrobial Activity of *Cissus quadrangularis* L.. J. Med. Food.

[73] Kim H., Kim C., Jhon G., Moon I., Choi S., Cho K., Chai J., Kim C. (2002). The Effect of Safflower Seed Extract on Periodontal Healing of 1-Wall Intrabony Defects in Beagle Dogs. J. Periodontol..

[74] Dobrin A., Popa G. (2022). Evaluation of the Antimicrobial Activity of Carthamus Tinctorius Extracts Against Nosocomial Microorganisms. Sci. Papers Ser. B Hortic..

[75] Merolli A., Nicolais L., Ambrosio L., Santin M. (2010). A degradable soybean-based biomaterial used effectively as a bone filler in vivo in a rabbit. Biomed. Mater..

[76] Chaleshtori S.A.H., Kachoie M.A., Jazi S.M.H. (2017). Antibacterial effects of the methanolic extract of *Glycine max* (Soybean). Microbiol. Res..

[77] Agrawal A., Reche A., Agrawal S., Paul P. (2023). Applications of Chitosan Nanoparticles in Dentistry: A Review. Cureus.

[78] Ke C.-L., Deng F.-S., Chuang C.-Y., Lin C.-H. (2021). Antimicrobial Actions and Applications of Chitosan. Polymers.

[79] Lo W.-H., Deng F.-S., Chang C.-J., Lin C.-H. (2020). Synergistic Antifungal Activity of Chitosan with Fluconazole against *Candida albicans*, *Candida tropicalis*, and Fluconazole-Resistant Strains. Molecules.

[80] Tahsin K., Xu W., Watson D., Rizkalla A., Charpentier P. (2025). Antimicrobial Denture Material Synthesized from Poly(methyl methacrylate) Enriched with Cannabidiol Isolates. Molecules.

[81] Beyth N., Yudovin-Farber I., Bahir R., Domb A.J., Weiss E.I. (2006). Antibacterial activity of dental composites containing quaternary ammonium polyethylenimine nanoparticles against *Streptococcus mutans*. Biomaterials.

[82] Xu X., Wang Y., Liao S., Wen Z.T., Fan Y. (2012). Synthesis and characterization of antibacterial dental monomers and composites. J. Biomed. Mater. Res. B Appl. Biomater..

[83] Xu X., Costin S. (2013). Chapter 10. Antimicrobial Polymeric Dental Materials. Polymeric Materials with Antimicrobial Activity: From Synthesis to Applications.

[84] Imazato S., Imai T., Russell R.R.B., Torii M., Ebisu S. (1998). Antibacterial activity of cured dental resin incorporating the antibacterial monomer MDPB and an adhesion-promoting monomer. J. Biomed. Mater. Res..

[85] Imazato S., Torii M., Tsuchitani Y., McCabe J.F., Russell R.R.B. (1994). Incorporation of Bacterial Inhibitor into Resin Composite. J. Dent. Res..

[86] Ge Y., Wang S., Zhou X., Wang H., Xu H., Cheng L. (2015). The Use of Quaternary Ammonium to Combat Dental Caries. Materials.

[87] Chen L., Suh B.I., Yang J. (2018). Antibacterial dental restorative materials: A review. Am. J. Dent..

[88] US Food and Drug Admistration (1985). Sec. 172.165 Quaternary Ammonium Chloride Combination. https://www.accessdata.fda.gov/scripts/cdrh/cfdocs/cfcfr/CFRSearch.cfm?fr=172.165.

[89] Zhou Z., Zhou S., Zhang X., Zeng S., Xu Y., Nie W., Zhou Y., Xu T., Chen P. (2023). Quaternary Ammonium Salts: Insights into Synthesis and New Directions in Antibacterial Applications. Bioconjug. Chem..

[90] Ioannou C.J., Hanlon G.W., Denyer S.P. (2007). Action of Disinfectant Quaternary Ammonium Compounds against *Staphylococcus aureus*. Antimicrob. Agents Chemother..

[91] Luz A., DeLeo P., Pechacek N., Freemantle M. (2020). Human health hazard assessment of quaternary ammonium compounds: Didecyl dimethyl ammonium chloride and alkyl (C12–C16) dimethyl benzyl ammonium chloride. Regul. Toxicol. Pharmacol..

[92] Ye X., Qin X., Yan X., Guo J., Huang L., Chen D., Wu T., Shi Q., Tan S., Cai X. (2016). π–π conjugations improve the long-term antibacterial properties of graphene oxide/quaternary ammonium salt nanocomposites. Chem. Eng. J..

[93] Padnya P.L., Terenteva O., Akhmedov A., Iksanova A., Shtyrlin N., Nikitina E., Krylova E., Shtyrlin Y.G., Stoikov I. (2021). Thiacalixarene based quaternary ammonium salts as promising antibacterial agents. Bioorg. Med. Chem..

[94] Zhang X., Kong H., Zhang X., Jia H., Ma X., Miao H., Mu Y., Zhang G. (2021). Design and production of environmentally degradable quaternary ammonium salts. Green Chem..

[95] Benkova M., Soukup O., Prchal L., Sleha R., Eleršek T., Novak M., Sepčić K., Gunde-Cimerman N., Dolezal R., Bostik V. (2019). Synthesis, Antimicrobial Effect and Lipophilicity-Activity Dependence of Three Series of Dichained *N*-Alkylammonium Salts. ChemistrySelect.

[96] Zhi L., Shi X., Zhang E., Gao C., Gai H., Wang H., Liu Z., Zhang T. (2022). Synthesis and Performance of Double-Chain Quaternary Ammonium Salt Glucosamide Surfactants. Molecules.

[97] Murata H., Koepsel R.R., Matyjaszewski K., Russell A.J. (2007). Permanent, non-leaching antibacterial surfaces—2: How high density cationic surfaces kill bacterial cells. Biomaterials.

[98] Xie D., Weng Y., Guo X., Zhao J., Gregory R.L., Zheng C. (2011). Preparation and evaluation of a novel glass-ionomer cement with antibacterial functions. Dent. Mater..

[99] Cheng L., Weir M.D., Zhang K., Arola D.D., Zhou X., Xu H.H.K. (2013). Dental primer and adhesive containing a new antibacterial quaternary ammonium monomer dimethylaminododecyl methacrylate. J. Dent..

[100] Zhou C., Weir M.D., Zhang K., Deng D., Cheng L., Xu H.H.K. (2013). Synthesis of new antibacterial quaternary ammonium monomer for incorporation into CaP nanocomposite. Dent. Mater..

[101] Melo M.A.S., Wu J., Weir M.D., Xu H.H.K. (2014). Novel antibacterial orthodontic cement containing quaternary ammonium monomer dimethylaminododecyl methacrylate. J. Dent..

[102] Li F., Weir M.D., Fouad A.F., Xu H.H.K. (2014). Effect of salivary pellicle on antibacterial activity of novel antibacterial dental adhesives using a dental plaque microcosm biofilm model. Dent. Mater..

[103] Wang S., Zhang K., Zhou X., Xu N., Xu H.H.K., Weir M.D., Ge Y., Wang S., Li M., Li Y. (2014). Antibacterial Effect of Dental Adhesive Containing Dimethylaminododecyl Methacrylate on the Development of *Streptococcus mutans* Biofilm. Int. J. Mol. Sci..

[104] Zhang K., Wang S., Zhou X., Xu H., Weir M.D., Ge Y., Li M., Li Y., Xu X., Zheng L. (2015). Effect of Antibacterial Dental Adhesive on Multispecies Biofilms Formation. J. Dent. Res..

[105] Marsh P.D., Bradshaw D.J. (1995). Dental plaque as a biofilm. J. Ind. Microbiol..

[106] Pashley D.H., Tay F., Yiu C., Hashimoto M., Breschi L., Carvalho R., Ito S. (2004). Collagen Degradation by Host-derived Enzymes during Aging. J. Dent. Res..

[107] Chen C., Weir M.D., Cheng L., Lin N.J., Lin-Gibson S., Chow L.C., Zhou X., Xu H.H. (2014). Antibacterial activity and ion release of bonding agent containing amorphous calcium phosphate nanoparticles. Dent. Mater..

[108] Zhang K., Cheng L., Wu E.J., Weir M.D., Bai Y., Xu H.H.K. (2013). Effect of water-ageing on dentine bond strength and anti-biofilm activity of bonding agent containing new monomer dimethylaminododecyl methacrylate. J. Dent..

[109] Li F., Weir M.D., Xu H.H.K. (2013). Effects of Quaternary Ammonium Chain Length on Antibacterial Bonding Agents. J. Dent. Res..

[110] Li F., Weir M.D., Fouad A.F., Xu H.H.K. (2013). Time-kill behaviour against eight bacterial species and cytotoxicity of antibacterial monomers. J. Dent..

[111] Li F., Wang P., Weir M.D., Fouad A.F., Xu H.H.K. (2014). Evaluation of antibacterial and remineralizing nanocomposite and adhesive in rat tooth cavity model. Acta Biomater..

[112] Zhou H., Li F., Weir M.D., Xu H.H.K. (2013). Dental plaque microcosm response to bonding agents containing quaternary ammonium methacrylates with different chain lengths and charge densities. J. Dent..

[113] Li F., Weir M.D., Chen J., Xu H.H.K. (2014). Effect of charge density of bonding agent containing a new quaternary ammonium methacrylate on antibacterial and bonding properties. Dent. Mater..

[114] Zhang N., Ma J., Melo M.A.S., Weir M.D., Bai Y., Xu H.H.K. (2015). Protein-repellent and antibacterial dental composite to inhibit biofilms and caries. J. Dent..

[115] Karakullukcu A.B., Taban E., Ojo O.O. (2023). Biocompatibility of biomaterials and test methods: A review. Mater. Test..

[116] Al-Shalawi F.D., Ariff A.H.M., Jung D.-W., Ariffin M.K.A.M., Kim C.L.S., Brabazon D., Al-Osaimi M.O. (2023). Biomaterials as Implants in the Orthopedic Field for Regenerative Medicine: Metal versus Synthetic Polymers. Polymers.

[117] Binlateh T., Thammanichanon P., Rittipakorn P., Thinsathid N., Jitprasertwong P. (2022). Collagen-Based Biomaterials in Periodontal Regeneration: Current Applications and Future Perspectives of Plant-Based Collagen. Biomimetics.

[118] Talha M., Behera C.K., Sinha O.P. (2013). A review on nickel-free nitrogen containing austenitic stainless steels for biomedical applications. Mater. Sci. Eng. C.

[119] Niinomi M. (1998). Mechanical properties of biomedical titanium alloys. Mater. Sci. Eng. A.

[120] Zhang F., Cai S., Xu G., Shen S., Li Y., Zhang M., Wu X. (2016). Corrosion behavior of mesoporous bioglass-ceramic coated magnesium alloy under applied forces. J. Mech. Behav. Biomed. Mater..

[121] Wang H.-Y., Zhu R.-F., Lu Y.-P., Xiao G.-Y., He K., Yuan Y., Ma X.-N., Li Y. (2014). Effect of sandblasting intensity on microstructures and properties of pure titanium micro-arc oxidation coatings in an optimized composite technique. Appl. Surf. Sci..

[122] Civantos A., Martínez-Campos E., Ramos V., Elvira C., Gallardo A., Abarrategi A. (2017). Titanium Coatings and Surface Modifications: Toward Clinically Useful Bioactive Implants. ACS Biomater. Sci. Eng..

[123] Catauro M., Bollino F., Papale F. (2014). Biocompatibility improvement of titanium implants by coating with hybrid materials synthesized by sol-gel technique. J. Biomed. Mater. Res. A.

[124] Midha S., Kim T.B., Van Den Bergh W., Lee P.D., Jones J.R., Mitchell C.A. (2013). Preconditioned 70S30C bioactive glass foams promote osteogenesis in vivo. Acta Biomater..

[125] Niinomi M. (2002). Recent metallic materials for biomedical applications. Metall. Mater. Trans. A.

[126] Kickelbick G. (2006). Introduction to Hybrid Materials. Hybrid Materials.

[127] Novak B.M. (1993). Hybrid Nanocomposite Materials—Between inorganic glasses and organic polymers. Adv. Mater..

[128] Grosso D., Ribot F., Boissiere C., Sanchez C. (2011). Molecular and supramolecular dynamics of hybrid organic–inorganic interfaces for the rational construction of advanced hybrid nanomaterials. Chem. Soc. Rev..

[129] Sanchez C., Julián B., Belleville P., Popall M. (2005). Applications of hybrid organic–inorganic nanocomposites. J. Mater. Chem..

[130] Khurshid Z., Husain S., Alotaibi H., Rehman R., Zafar M.S., Farooq I., Khan A.S. (2019). Novel Techniques of Scaffold Fabrication for Bioactive Glasses. Biomedical, Therapeutic and Clinical Applications of Bioactive Glasses.

[131] Dong H., Liu H., Zhou N., Li Q., Yang G., Chen L., Mou Y. (2020). Surface Modified Techniques and Emerging Functional Coating of Dental Implants. Coatings.

[132] Habibah T.U., Amlani D.V., Brizuela M. (2025). Hydroxyapatite Dental Material.

[133] Islam M.A., Hossain N., Hossain S., Khan F., Hossain S., Arup M.R., Chowdhury M.A., Rahman M. (2025). Advances of Hydroxyapatite Nanoparticles in Dental Implant Applications. Int. Dent. J..

[134] Al-Noaman A., Rawlinson S.C.F., Hill R.G. (2022). MgF_2_-containing glasses as a coating for titanium dental implant. I- Glass powder. J. Mech. Behav. Biomed. Mater..

[135] Oliver J.A.N., Su Y., Lu X., Kuo P.H., Du J., Zhu D. (2019). Bioactive glass coatings on metallic implants for biomedical applications. Bioact. Mater..

[136] Rohr N., Nebe J.B., Schmidli F., Müller P., Weber M., Fischer H., Fischer J. (2019). Influence of bioactive glass-coating of zirconia implant surfaces on human osteoblast behavior in vitro. Dent. Mater..

[137] Zhang M., Pu X., Chen X., Yin G. (2019). In-vivo performance of plasma-sprayed CaO–MgO–SiO_2_-based bioactive glass-ceramic coating on Ti–6Al–4V alloy for bone regeneration. Heliyon.

[138] Hu D., Li K., Xie Y., Pan H., Zhao J., Huang L., Zheng X. (2016). Different response of osteoblastic cells to Mg^2+^, Zn^2+^ and Sr^2+^ doped calcium silicate coatings. J. Mater. Sci. Mater. Med..

[139] Kinnunen I., Aitasalo K., Pöllönen M., Varpula M. (2000). Reconstruction of orbital floor fractures using bioactive glass. J. Cranio-Maxillofac. Surg..

[140] Zafar M.S., Farooq I., Awais M., Najeeb S., Khurshid Z., Zohaib S. (2019). Bioactive Surface Coatings for Enhancing Osseointegration of Dental Implants. Biomedical, Therapeutic and Clinical Applications of Bioactive Glasses.

[141] Yoshimura M., Suda H., Okamoto K., Ioku K. (1994). Hydrothermal synthesis of biocompatible whiskers. J. Mater. Sci..

[142] Silva C.C., Pinheiro A.G., Miranda M.A.R., Góes J.C., Sombra A.S.B. (2003). Structural properties of hydroxyapatite obtained by mechanosynthesis. Solid State Sci..

[143] Saeri M.R., Afshar A., Ghorbani M., Ehsani N., Sorrell C.C. (2003). The wet precipitation process of hydroxyapatite. Mater. Lett..

[144] Shih W.-J., Chen Y.-F., Wang M.-C., Hon M.-H. (2004). Crystal growth and morphology of the nano-sized hydroxyapatite powders synthesized from CaHPO_4_·2H_2_O and CaCO_3_ by hydrolysis method. J. Cryst. Growth.

[145] Kim I.-S., Kumta P.N. (2004). Sol–gel synthesis and characterization of nanostructured hydroxyapatite powder. Mater. Sci. Eng. B.

[146] Pandey S., Mishra S.B. (2011). Sol–gel derived organic–inorganic hybrid materials: Synthesis, characterizations and applications. J. Solgel Sci. Technol..

[147] Hench L.L., West J.K. (1990). The sol-gel process. Chem. Rev..

[148] Al Zoubi W., Kamil M.P., Fatimah S., Nisa N., Ko Y.G. (2020). Recent advances in hybrid organic-inorganic materials with spatial architecture for state-of-the-art applications. Prog. Mater. Sci..

[149] Jones J.R. (2013). Review of bioactive glass: From Hench to hybrids. Acta Biomater..

[150] Mondal D., Dixon S.J., Mequanint K., Rizkalla A.S. (2018). Bioactivity, Degradation, and Mechanical Properties of Poly(vinylpyrrolidone-*co*-triethoxyvinylsilane)/Tertiary Bioactive Glass Hybrids. ACS Appl. Bio Mater..

[151] Cinici B., Yaba S., Kurt M., Yalcin H.C., Duta L., Gunduz O. (2024). Fabrication Strategies for Bioceramic Scaffolds in Bone Tissue Engineering with Generative Design Applications. Biomimetics.

[152] Gao Y., Seles M.A., Rajan M. (2023). Role of bioglass derivatives in tissue regeneration and repair: A review. Rev. Adv. Mater. Sci..

[153] Tshikovhi A., Koao L.F., Malevu T.D., Linganiso E.C., Motaung T.E. (2023). Dopants concentration on the properties of various host materials by sol-gel method: Critical review. Results Mater..

[154] Tinoco Navarro L.K., Jaroslav C. (2023). Enhancing Photocatalytic Properties of TiO2 Photocatalyst and Heterojunctions: A Comprehensive Review of the Impact of Biphasic Systems in Aerogels and Xerogels Synthesis, Methods, and Mechanisms for Environmental Applications. Gels.

[155] Barrino F. (2024). Hybrid Organic–Inorganic Materials Prepared by Sol–Gel and Sol–Gel-Coating Method for Biomedical Use: Study and Synthetic Review of Synthesis and Properties. Coatings.

[156] Zvonkina I., Soucek M. (2016). Inorganic–organic hybrid coatings: Common and new approaches. Curr. Opin. Chem. Eng..

[157] Tranquillo E., Bollino F. (2020). Surface Modifications for Implants Lifetime extension: An Overview of Sol-Gel Coatings. Coatings.

[158] Catauro M., Barrino F., Bononi M., Colombini E., Giovanardi R., Veronesi P., Tranquillo E. (2019). Coating of Titanium Substrates with ZrO_2_ and ZrO_2_-SiO_2_ Composites by Sol-Gel Synthesis for Biomedical Applications: Structural Characterization, Mechanical and Corrosive Behavior. Coatings.

[159] Mendhe A.C. (2023). Spin Coating: Easy Technique for Thin Films. Simple Chemical Methods for Thin Film Deposition: Synthesis and Applications.

[160] Gvishi R., Sokolov I. (2020). 3D sol–gel printing and sol–gel bonding for fabrication of macro- and micro/nano-structured photonic devices. J. Solgel Sci. Technol..

[161] Kawashita M., Tsuneyama S., Miyaji F., Kokubo T., Kozuka H., Yamamoto K. (2000). Antibacterial silver-containing silica glass prepared by sol–gel method. Biomaterials.

[162] Bachvarova-Nedelcheva A., Kostova Y., Yordanova L., Nenova E., Shestakova P., Ivanova I., Pavlova E. (2024). Sol–Gel Synthesis of Silica–Poly (Vinylpyrrolidone) Hybrids with Prooxidant Activity and Antibacterial Properties. Molecules.

[163] Bryaskova R., Pencheva D., Kale G.M., Lad U., Kantardjiev T. (2010). Synthesis, characterisation and antibacterial activity of PVA/TEOS/Ag-Np hybrid thin films. J. Colloid Interface Sci..

[164] Copello G.J., Teves S., Degrossi J., D’Aquino M., Desimone M.F., Diaz L.E. (2006). Antimicrobial activity on glass materials subject to disinfectant xerogel coating. J. Ind. Microbiol. Biotechnol..

[165] Nablo B.J., Rothrock A.R., Schoenfisch M.H. (2005). Nitric oxide-releasing sol–gels as antibacterial coatings for orthopedic implants. Biomaterials.

[166] Ubhale Y.S., More A.P. (2024). Antimicrobial sol–gel coating: A review. J. Coat. Technol. Res..

[167] Gunawidjaja P.N., Lo A.Y.H., Izquierdo-Barba I., García A., Arcos D., Stevensson B., Grins J., Vallet-Regí M., Edén M. (2010). Biomimetic Apatite Mineralization Mechanisms of Mesoporous Bioactive Glasses as Probed by Multinuclear ^31^P, ^29^Si, ^23^Na and ^13^C Solid-State NMR. J. Phys. Chem. C.

[168] Hench L.L. (2013). Chronology of Bioactive Glass Development and Clinical Applications. New J. Glass Ceram..

[169] Skallevold H.E., Rokaya D., Khurshid Z., Zafar M.S. (2019). Bioactive Glass Applications in Dentistry. Int. J. Mol. Sci..

[170] Moreno M.G., Butini M.E., Maiolo E.M., Sessa L., Trampuz A. (2020). Antimicrobial activity of bioactive glass S53P4 against representative microorganisms causing osteomyelitis—Real-time assessment by isothermal microcalorimetry. Colloids Surf. B Biointerfaces.

[171] (2009). Biological Evaluation of Medical Devices Part 5: Tests for In Vitro Cytotoxicity.

[172] (2021). Biological Evaluation of Medical Devices Part 10: Tests for Skin Sensitization.

[173] (2014). Biological Evaluation of Medical Devices Part 3: Tests for Genotoxicity, Carcinogenicity and Reproductive Toxicity.

[174] (2016). Biological Evaluation of Medical Devices Part 6: Tests for Local Effects after Implantation.

[175] (2017). Biological Evaluation of Medical Devices Part 11: Tests for Systemic Toxicity.

[176] (2020). Paints and Varnishes—Cross-Cut Test.

[177] (2022). Standard Test Method for Pull-Off Strength of Coatings Using Portable Adhesion Testers.

[178] (2016). Dentistry—Implants—Dynamic Loading Test for Endosseous Dental Implants.

[179] (2011). Measurement of Antibacterial Activity on Plastics and Other Non-Porous Surfaces.

[180] (2010). Antibacterial Products—Test for Antibacterial Activity and Efficacy.

[181] Jiao Y., Niu L.-N., Ma S., Li J., Tay F.R., Chen J.-H. (2017). Quaternary ammonium-based biomedical materials: State-of-the-art, toxicological aspects and antimicrobial resistance. Prog. Polym. Sci..

[182] Grabińska-Sota E. (2011). Genotoxicity and biodegradation of quaternary ammonium salts in aquatic environments. J. Hazard. Mater..

