# Supplementary material for: Antimicrobial Materials Used in Coating Dental Implant Surfaces: State of the Art and Future Prospectives
Source: Materials (Basel). 2026 Jan 19;19(2):403. doi: 10.3390/ma19020403 (PMC12843238; doi:10.3390/ma19020403)
Supplement: Supplementary file 1 [file materials-19-00403-s001.zip › materials-3874306-supplementary.pdf]

S1. Review Methodology

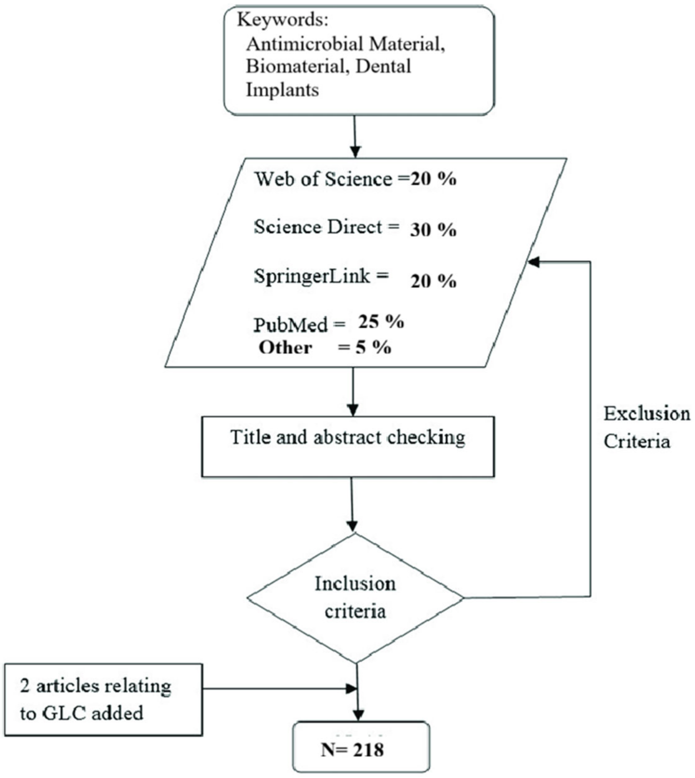

A comprehensive literature search was conducted in PubMed, Scopus, and Web of Science for articles published between January 2010 and June 2025 using the keywords "classifications of antimicrobial materials," "classification of biomaterials," and "dental materials." Only peer-reviewed articles written in English were included. Editorials, conference abstracts, and duplicate studies were excluded. Flow diagram of the systematic review is found in supplementary data.
